# The *syn*/*anti*-Dichotomy in the Palladium-Catalyzed Addition of Nucleophiles to Alkenes

**DOI:** 10.1002/chem.201404070

**Published:** 2014-11-05

**Authors:** Pavel Kočovský, Jan-E Bäckvall

**Affiliations:** [a] Department of Organic Chemistry, Arrhenius Laboratory, Stockholm University10691 Stockholm (Sweden); [b] Institute of Organic Chemistry and Biochemistry, Academy of Sciences of the Czech Republic, Department of Organic Chemistry, Charles UniversityFlemingovo nám. 2, 16610 Prague 6 (Czech Republic), Hlavova 8 12843 Prague 2 (Czech Republic)

**Keywords:** alkenes, catalysis, nucleophilic addition, palladium, stereochemistry

## Abstract

In this review the stereochemistry of palladium-catalyzed addition of nucleophiles to alkenes is discussed, and examples of these reactions in organic synthesis are given. Most of the reactions discussed involve oxygen and nitrogen nucleophiles; the Wacker oxidation of ethylene has been reviewed in detail. An *anti*-hydroxypalladation in the Wacker oxidation has strong support from both experimental and computational studies. From the reviewed material it is clear that *anti*-addition of oxygen and nitrogen nucleophiles is strongly favored in intermolecular addition to olefin–palladium complexes even if the nucleophile is coordinated to the metal. On the other hand, *syn*-addition is common in the case of intramolecular oxy- and amidopalladation as a result of the initial coordination of the internal nucleophile to the metal.

## 1. Introduction

**Scheme 1:**
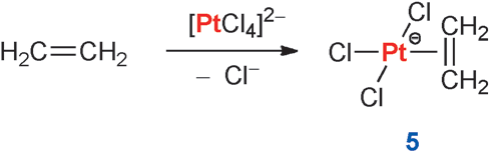
Addition of bromine and transition metals to alkenes.

Electrophilic addition to olefins is one of the fundamental reactions in organic chemistry. Thus, bromine or hypobromous acid are readily added across an electron-rich olefinic double bond (**1**) in a process that is initiated by an electrophilic attack to generate the corresponding bromonium ion **2**, which is then opened by Br^−^ (in the case of Br_2_) or H_2_O (when HOBr is employed) from the opposite side, to afford the *anti*-addition product (Scheme 1).[1,2] Electrophilic metal cations M^*n*+^, such as Hg^2+^ and Tl^3+^, follow a similar pattern in both inter- and intramolecular reactions.[1,3] By contrast, owing to the nature of the electron-rich C=C bonds, nucleophilic additions are rare and typically limited to intramolecular additions of an alkoxide moiety, generated from a suitable polycyclic alkenol by deprotonation with NaH or *t*BuOK.[4] Transition metals are known to form η^2^-complexes **3** that can be attacked by nucleophiles to effect a formal nucleophilic addition upon the removal of the metal.[5] However, the transition-metal-promoted process is more complicated, since the nucleophile can, a priori, attack the η^2^-species either from the opposite side (**3**) in analogy to bromonium ions **2** and their congeners, or could first coordinate to the metal and then be delivered in a *syn*-fashion to the original alkene (**4**).[5]

**Scheme 2:**
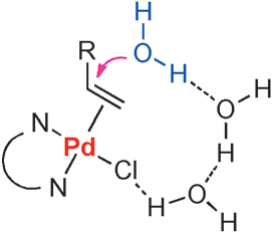
Preparation of Zeisse’s salt.

The first η^2^-olefin metal complex **5** was prepared by Zeise (Scheme 2) via coordination of ethylene (generated by an in situ dehydrogenation of boiling ethanol) to K_2_PtCl_4_.[6] However, this complex was long considered as a rarity and it took more than 100 years before the nature of the π bond between an alkene and a metal was understood and its potential had been realized.

The present review is concerned with the stereochemistry of nucleophilic additions to η^2^-alkene metal complexes of palladium (analogous to **5**) and application of these reactions in synthetic organic chemistry. The experimental and theoretical findings, accumulated over the years, are submitted to a thorough mechanistic analysis in light of the most recent results, which are not included in previous reviews. The new results have allowed us to draw conclusions that were not explicitly expressed in previous reviews.[7]

## 2. Wacker Oxidation

**Scheme 3:**
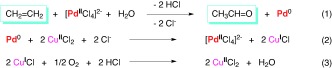
Wacker oxidation.

Apart from catalytic hydrogenation, the first large-scale industrial application of palladium was developed by Smidt and his colleagues at the Wacker company in the 1950s.[8] This process, which converts ethylene into acetaldehyde, is known as the Wacker oxidation (Scheme 3). Here, the tetrachloropalladate first generates the corresponding η^2^-ethylene-Pd complex (in analogy to the formation of Zeise’s salt **5**), which then reacts with water to produce acetaldehyde, Pd^0^, and two equivalents each of hydrogen chloride and chloride ions [Eq. (1)]. In order to make the process catalytic, the resulting Pd^0^ needs to be reoxidized to its active form, that is, Pd^II^, which is effected by the reaction with CuCl_2_ (2 equiv), using the two equivalents of Cl^−^ generated in the previous step [Eq. (2)]. The latter reaction produces CuCl, which is then reoxidized to CuCl_2_ by molecular oxygen, consuming the two equivalents of HCl generated in the first step [Eq. (3)]. The reaction is thus catalytic in both Pd^II^ and Cu^II^, with molecular oxygen serving as the terminal, stoichiometric oxidant. Note that direct reoxidation of Pd^0^ to Pd^II^ by molecular oxygen in water under Wacker conditions is too slow and would not prevent aggregation and formation of metallic palladium.

**Scheme 4:**
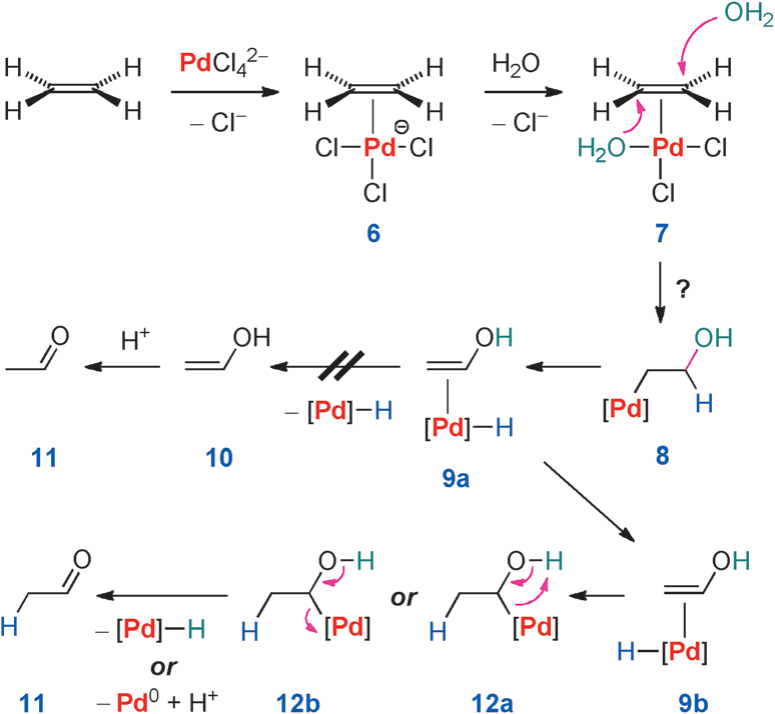
Wacker oxidation.

After the original publication on the Wacker process, this reaction became the subject of numerous mechanistic studies.[7a It was proposed that the intermediate η^2^-complex **7** reacts with water to produce the 2-hydroxyethylpalladium complex **8**, which would undergo a β-H elimination to generate vinyl alcohol **10** (via **9 a**), tautomerization of which would produce acetaldehyde **11** (Scheme 4). However, deuterium labeling studies clearly demonstrated that the final stages of the cascade do not adopt this route: the η^2^-complex **9 a**, primarily arising from **8** by the expected β-H elimination, does not dissociate to produce **10**, but rather undergoes an insertion reaction to generate complex **12** (via rotamer **9 b**), which now uses the O=H (rather than C=H) for the final β-H elimination (**12 a** or **12 b**) to produce acetaldehyde (**11**).[9] An alternative pathway, where a lone-pair on oxygen ejects Pd^0^ as a leaving group (**12 b**), was also considered,[10] which was later supported by theoretical calculations.[11]

**Scheme 5:**
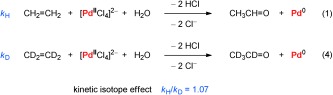
Deuterium labeling in Wacker oxidation.

The latter mechanism has been inferred from isotopic labeling as follows (Scheme 5): If the reaction proceeded through enol **10**, the molecule would abstract a proton from the environment [note that there is free HCl available, according to Equation (1). However, the experiment with perdeuteriated ethylene [Eq. (4)] showed that all the label was retained, which is consistent with the mechanism depicted in Scheme 4, namely with the isomerization **9 a** → **9 b** → **12**. The isotope effect [compare Eqs. (1) and (4) in Scheme 5] was found to be marginal.[9]

**Scheme 6:**
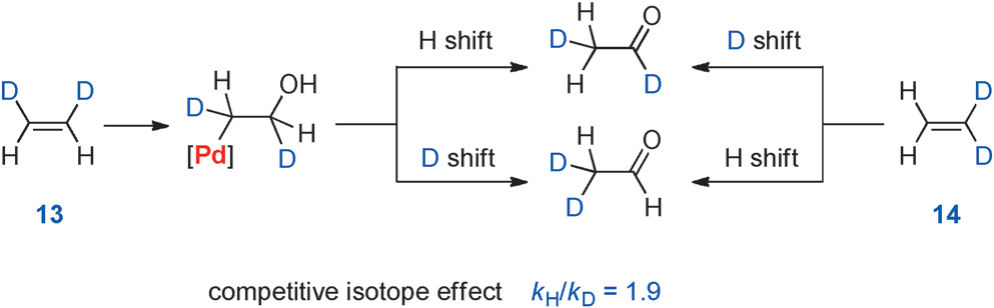
Isotope effect in Wacker oxidation.

In a complementary experiment, the specifically dideuteriated ethylenes **13** and **14** (Scheme 6) were found to exhibit an internal competitive isotope effect (H vs D shift), demonstrating that the rate-limiting step must occur before the β-H elimination and formation of acetaldehyde.[12,13] It was further argued that the rate-limiting step is the hydroxypalladation, and the results are better explained by a *syn*-migration since an *anti*-hydroxypalladation would seem to require a rate constant larger than diffusion control.[9,12]

**Scheme 7:**
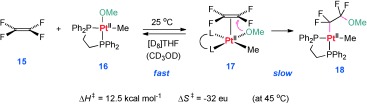
Experimentally observed *syn*-migration of MeO from platinum.

The proposed *syn*-migration of an oxygen nucleophile has been experimentally observed in a stoichiometric experiment with a Pt–olefin complex (Scheme 7). In this experiment the extremely electron-deficient tetrafluoroethylene (**15**) was treated with the Pt–OMe complex **16**. The formation of intermediate **17** and product **18**, as well as the kinetics, were monitored by NMR spectroscopy in [D_8_]THF with added CD_3_OD.[14] Here, an external attack by CD_3_OD (which should proceed with *anti* stereochemistry due to the steric hindrance from the Pt side) has not been observed, nor was the exchange of OCH_3_ with OCD_3_, so that the product **18** can only arise by the *syn*-migration of OCH_3_ from Pt. This may seem to support the *syn*-migration pathway in the Wacker process. However, it can be argued that the starting olefin **15** in this experiment is rather special, so that generalization of these findings, in particular to the Wacker oxidation of the electron-rich ethylene, cannot be made directly.

**Scheme 8:**
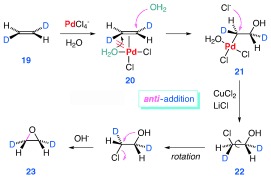
Stereochemistry of hydroxypalladation in the presence of chloride ions.

In order to elucidate the stereochemistry of hydroxypalladation, the *trans*-dideuteriated ethylene **19** was employed as a model compound (Scheme 8).[10,15] Under catalytic hydroxypalladation conditions, using a mixture of PdCl_2_, LiCl, and CuCl_2_, the latter alkene was expected to generate the η^2^-complex **20**, where the water molecule would have to be *cis*-coordinated toward the ethylene ligand (otherwise the *syn*-transfer would not be possible at all). The *anti*-attack by an external water molecule on **20** should generate complex **21**, which in the presence of chloride ions should produce chlorohydrin **22** as a result of the S_N_2 displacement of palladium. Treatment of **22** with a base would then produce epoxide **23**. The latter epoxide was found to be *cis*-configured (as shown), which is consistent with the pathway involving three inversions of configuration, that is, in each step starting with complex **20**. Since the replacement of palladium by Cl^−^ was known from the previous work[16] to proceed with inversion of configuration at carbon, and the stereochemistry of the chlorohydrin transformation into the corresponding epoxide is a textbook example of inversion, the key hydration **20** → **21** must also occur with inversion, that is, via an external attack as shown. This study thus provides evidence for the *anti*-addition mechanism for the key hydroxypalladation step.

**Scheme 9:**
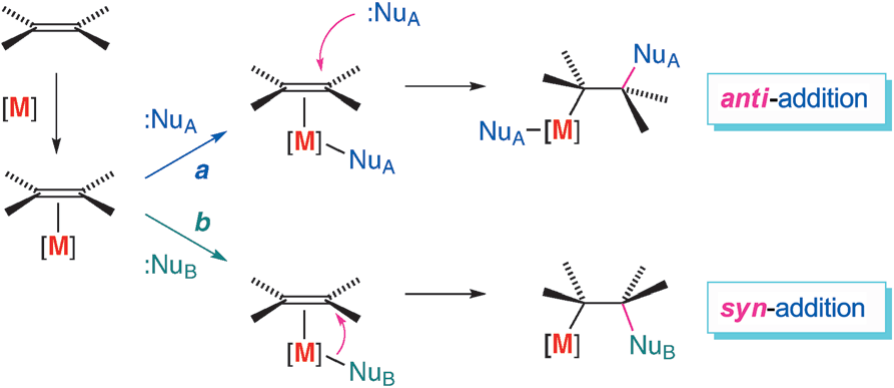
Stereochemistry of the reactions of metal complexes with nucleophiles.

An extended theoretical study[17] then reconciled the fact that depending on the nature of the nucleophile the nucleophilic attack may occur either in a *syn* or *anti* fashion (Scheme 9). Essentially, two types of nucleophiles can be discerned: Nu_A_, which can, a priori, coordinate the metal but prefer the external *anti*-addition to the olefinic ligand (pathway ***a***), and Nu_B_, which prefer an intramolecular, that is, *syn*-transfer (pathway ***b***).

**Figure 1:**
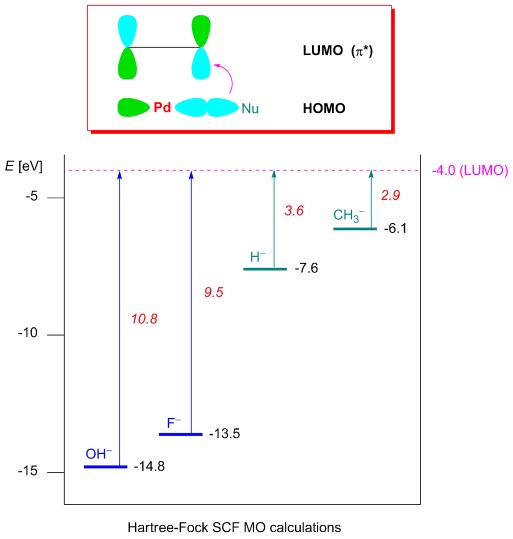
Orbital interactions in the palladium η^2^-complexes.

Analysis of the orbital interactions in four model systems, namely with OH^−^, F^−^, H^−^, and CH_3_^−^ as representative nucleophiles coordinated to Pd, revealed the following (Figure 1):[17b] the HOMO orbitals of the first two complexes lie rather low, so that their interaction with the LUMO of the (coordinated) olefin will be weak and result in little stabilization. By contrast, the two latter complexes have high energy HOMO orbitals for the Pd=Nu bond (Nu = H^−^ and CH_3_^−^), so that the interaction between the HOMO orbital of these complexes and the LUMO of the alkene should be stronger and lead to a considerable lowering of the energy of the system. Hence, it can be anticipated that the reactivity of H^−^ and CH_3_^−^ will be controlled mainly by orbital interactions, so that the transfer from the metal should be preferred. On the other hand, the orbital effects in the case of OH^−^ and F^−^ are likely to be weak, so that the reaction should be dominated by ionic effects and thus proceed via *anti*-addition.[17b]1

**Scheme 10:**
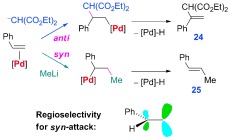
Regioselectivity as a result of the reaction stereochemistry.

Furthermore, the reactions controlled by ionic interaction (favoring the *anti*-pathway) can be expected to obey the Markovnikov rule like any other electrophilic addition and afford products, where the incoming nucleophile is planted on the carbon that can better stabilize a partial positive charge (**24** in Scheme 10). On the other hand, in the orbital-controlled reactions (favoring the *syn*-pathway) the nucleophile should add to the less-substituted carbon (**25**), as the LUMO orbital at that carbon should be larger.[17]

**Figure 2:**
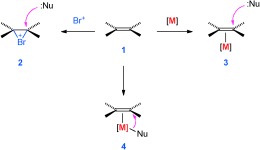
Calculated supramolecular interaction of water with the η^2^-complex.

A possible supramolecular interaction, involving several molecules of water and hydrogen bonding to the chloride bound to Pd, has also been considered for the Wacker oxidation (Figure 2).[18]

It has been argued that the chloride concentration in the experiments shown in Scheme 8 is higher (3 m) than in the Wacker process and in the kinetic experiments[9] (≤1 m) and that the stereochemistry may not be the same at the different concentrations of Cl^−^.[7,19,20] However, the chemistry of 1,4-difunctionalization of dienes, catalyzed by Pd(OAc)_2_, shows that while under chloride-free conditions, the acetate group is delivered preferentially to the intermediate allyl group from Pd (i.e., in the *syn*-fashion), addition of only catalytic amounts of LiCl (4 equiv per Pd) shuts down this reaction pathway. The chloride is replacing coordinated acetate on Pd, and in this way acetate can only be delivered in an *anti*-fashion by an intermolecular reaction.[21] Further increase of the chloride concentration does not alter the latter stereochemistry and only increases the proportion of the product resulting from the Cl^−^ attack on the η^3^-complex (rather than AcO^−^ attack). This is in direct analogy to the formation of chlorohydrin **22** in the Wacker process, lending additional support for the argument that the stereochemistry should be the same at high and moderate concentrations of chloride ions.

**Scheme 11:**
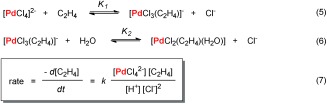
Chloride effect: rate inhibition.

**Scheme 12:**
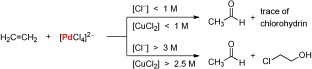
Chloride effect: change of mechanism and product distribution.

Kinetic studies have demonstrated that the Wacker oxidation is actually inhibited by an increasing concentration of chloride ions [Eqs. (5)–(7) in Scheme 11].^[7**,** 17**,** 19]^ In fact, at ≤1 m concentration of Cl^−^ and CuCl_2_ (industrial conditions for Wacker oxidation), acetaldehyde is the predominantly formed product (with small amounts of the corresponding chlorohydrin). By contrast, at 3 m concentration of Cl^−^ (conditions used in the mechanistic study shown in Scheme 8[15]), chlorohydrin becomes the predominant product (Scheme 12). It is highly unlikely that a change of the chloride ion concentration from 1 m to 3 m would change the stereochemistry of the hydroxypalladation of ethylene.

**Scheme 13:**
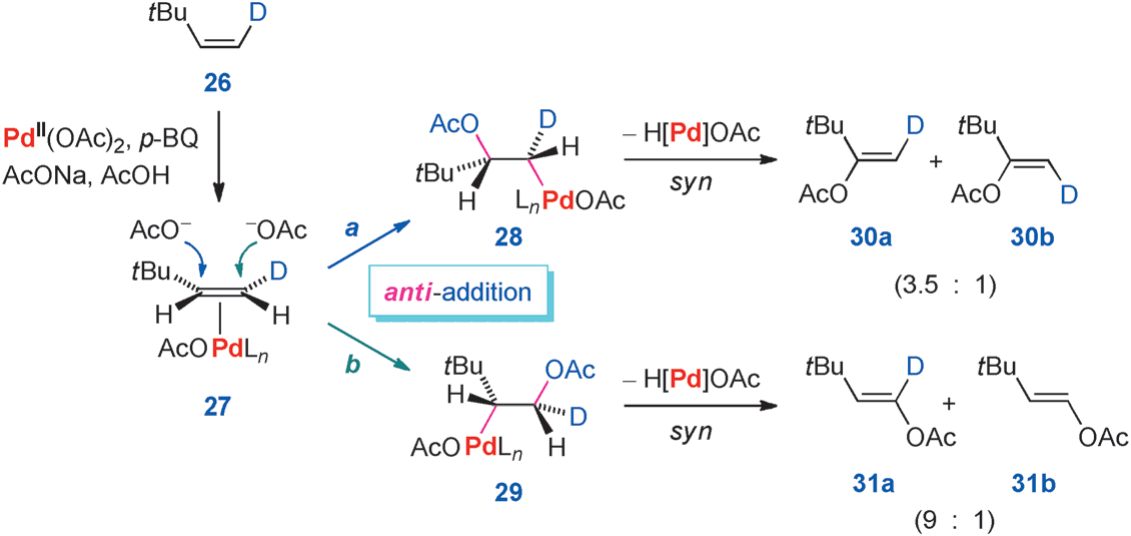
Stereochemistry of acetoxypalladation under chloride-free conditions.

Finally, strong support for the *anti*-addition of AcO^−^ and Pd^II^ under the chloride-free conditions has been obtained from the acetoxypalladation of deuteriated 3,3-dimethyl-1-butene **26** (Scheme 13).[22] Here, the isomeric products **30** and **31** were shown to arise from predominant *anti*-attack of AcO^−^ on the η^2^-Pd complex **27** (via the Markovnikov pathway ***a*** and anti-Markovnikov route ***b***), followed by the stereospecific β-hydrogen elimination from the respective intermediates **28** and **29** that occurs solely in a *syn*-fashion. Some loss of the stereochemical integrity observed for this process was attributed to a partial isomerization of the Markovnikov product (confirmed by a control experiment), so that the (*E*)- and (*Z*)-vinyl acetates **30 a** and **30 b** were obtained as a 3.5:1 mixture. The anti-Markovnikov product was obtained as a 9:1 mixture of (*E*)-isomers **31 a** and **31 b**. Here, the control experiment with non-deuteriated 3,3-dimethyl-1-butene showed that the Markovnikov product was a pure (*E*)-isomer, apparently arising from a conformation of the non-deuteriated congener of **29**, where the bulky *t*Bu and AcO groups avoid the *gauche* interaction. The 9:1 ratio of **31 a** and **31 b** thus suggests that the initial acetoxypalladation proceeds mainly (but not solely) via the *anti*-mechanism. Complementary results were obtained with the (*E*)-isomer of **26**.[22]

**Scheme 14:**
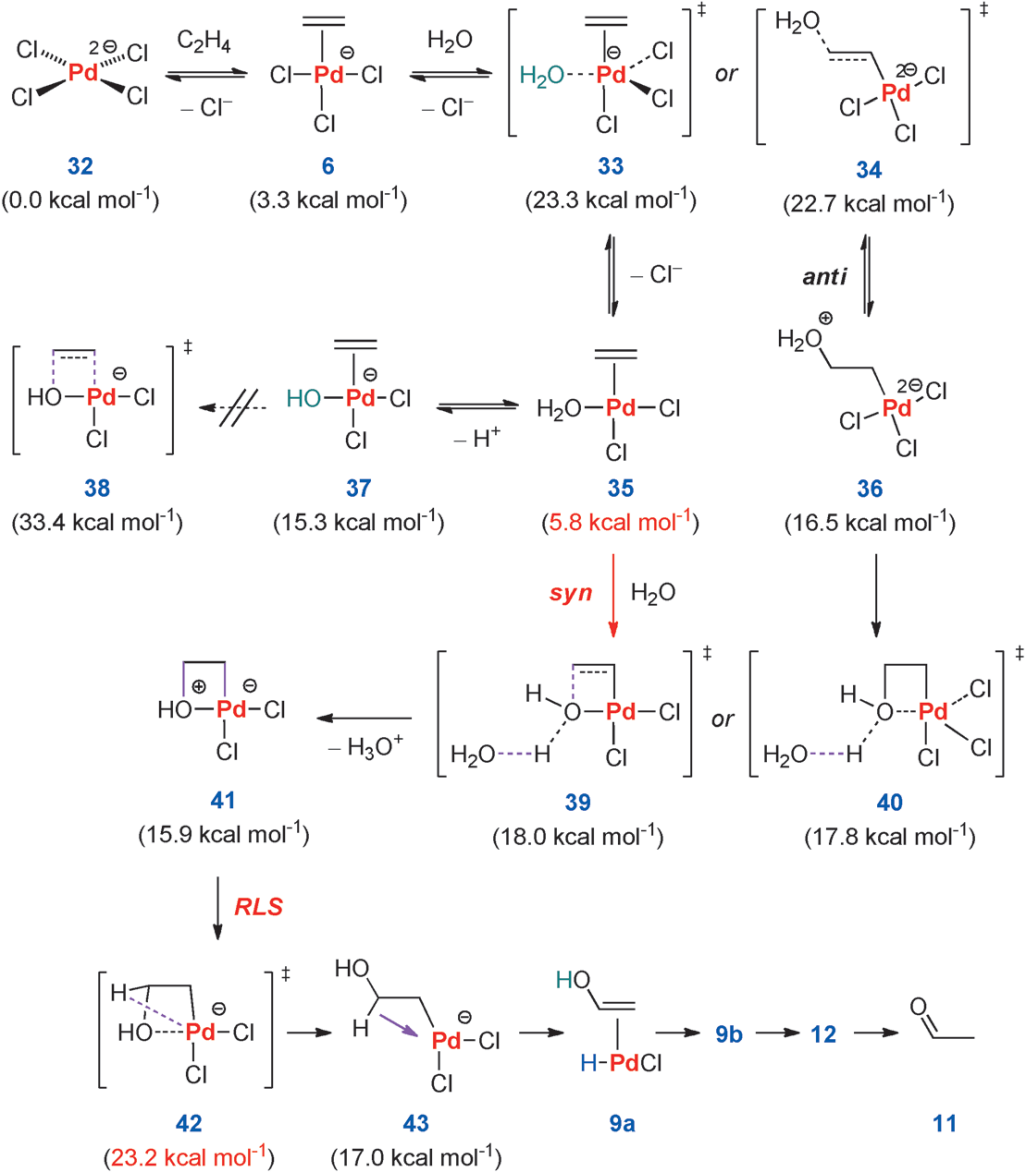
Energies of various intermediates in the proposed *syn*-mechanism of the Wacker oxidation in the absence of CuCl_2_.

In a computational study,[11] the energy of various intermediates and transition states that can be considered for the hydration pathway was assessed (Scheme 14). For the scenario with low concentration of Cl^−^, the starting tetrachloropalladate **32** is first converted into the η^2^-complex **6**, which can be attacked by water either at Pd or at the alkene ligand. The energy of the corresponding transition states **33** and **34** has been calculated to be almost identical but the Pd-hydrated product **35**, arising from the former TS^≠^, is by 10.7 kcal mol^−1^ lower in energy than **36**, arising from the latter TS^≠^. Deprotonation of **35** to generate **37**, which in principle could transfer the hydroxy group to the alkene ligand, would proceed through the transition state **38** but this species is rather high in energy (33.4 kcal mol^−1^), well above the experimentally overall observed value (22.4 kcal mol^−1^).

However, an alternative pathway, namely the water-assisted deprotonation of **35** with concomitant *syn*-migration, eventually leading to **41**, would proceed through the transition state **39** that is half-way down on the energy scale compared to **38**, indicating that this pathway would be favored if the *syn* addition mechanism operated. Conversely, the *anti*-delivery of water (**34**) generates intermediate **36**. Either of the transition states (**39** or **40**) would give rise to **41** by water-assisted deprotonation. Dissociation of the Pd=O bond in **41** would generate complex **43** ready for the expected β-H elimination. The calculations further found the energy of the corresponding transition state **42** (including the agostic Pd-H interaction) to be 23.2 kcal mol^−1^, which makes this species highest in energy in the whole cascade (via **39/40**),[11] so that this step can be regarded as rate-limiting.

However, there is a problem with this calculation since the transition state for the *anti-*attack is calculated on the negatively charged species **6**. The kinetics of the Wacker process shows that two chlorides are displaced, so an *anti*-attack would occur on the neutral species PdCl_2_(OH_2_)(CH_2_=CH_2_). It can be expected that the latter neutral species reacts much faster than the negatively charged species **6** in external *anti*-attack by water. This has also been confirmed later in other calculations (vide infra).

Subsequent calculations[23] suggested that the *anti*-addition mechanism is strongly favored over the *syn* pathway; this was disputed in a subsequent article[24] and the quality of the calculation was questioned.

**Scheme 15:**
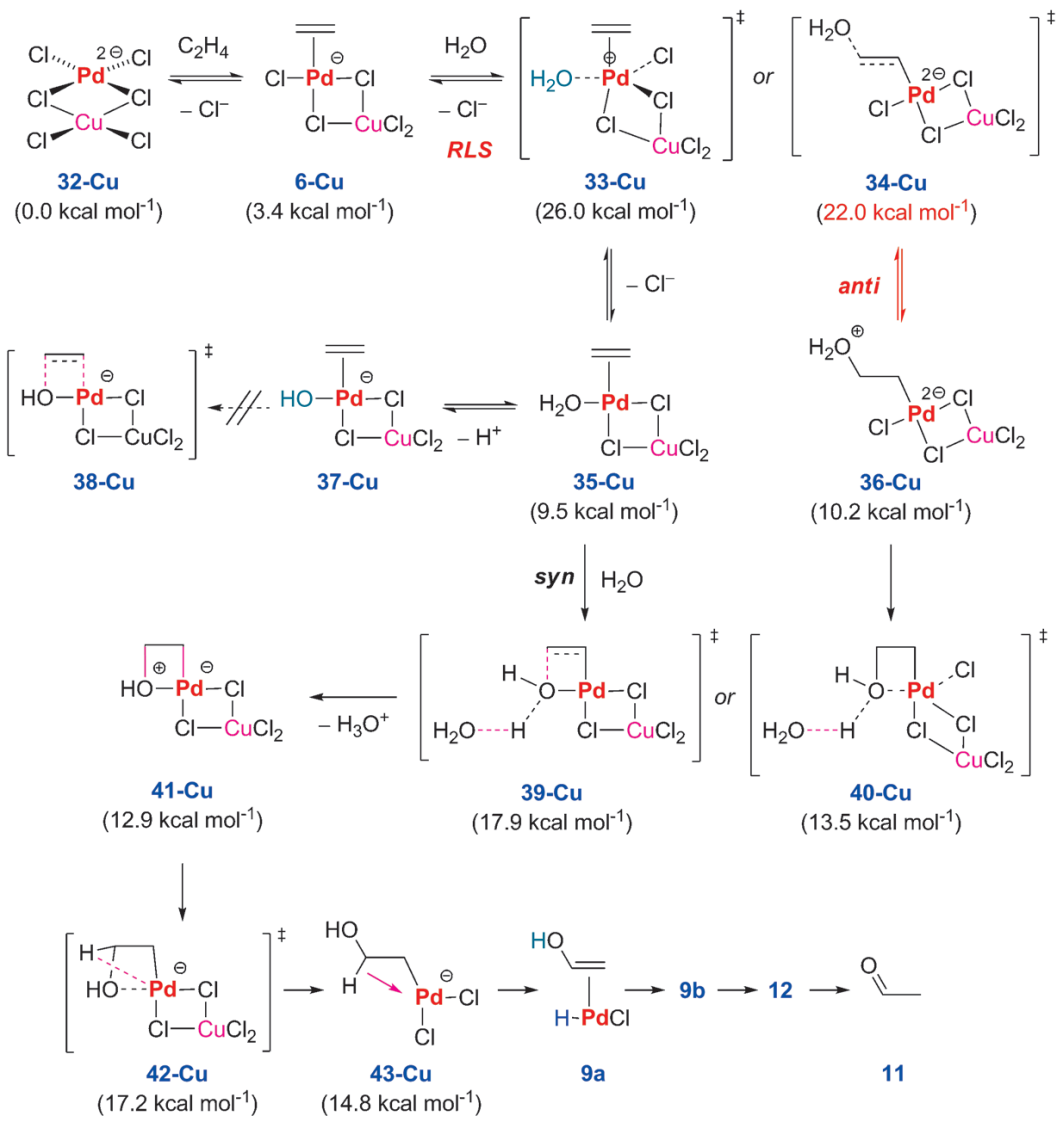
Energies of various intermediates in the proposed *syn*-mechanism of the Wacker oxidation in the presence of CuCl_2_.

Calculations including CuCl_2_ gave a slightly different picture,[11] as shown in Scheme 15 (the structures here bear the same numbers as in Scheme 14 but with added “Cu” so that a direct comparison can be made). Here, the initial PdCl_2_/CuCl_2_ complex **32-Cu** first coordinates ethylene to produce **6-Cu** (upon a loss of Cl^−^), which then can be attacked by water either at Pd or at the carbon via transition states **33-Cu** and **34-Cu**, respectively, of which the latter is lower in energy by 4.0 kcal mol^−1^, indicating that the *anti*-pathway is preferred. Furthermore, the energy of the TS^≠^ **33-Cu** is 26.0 kcal mol^−1^, which is slightly higher than the experimentally observed value (22.4 kcal mol^−1^). The two transition states would generate the hydrated species **35-Cu** and **36-Cu** that are almost equal in energy but the subsequent gradual conversion into **41-Cu** should preferably proceed through TS^≠^ **40-Cu** that is by 4.4 kcal mol^−1^ lower in energy than its congener **39-Cu**. The end-game from **41-Cu** via **42-Cu** and **43-Cu** has been calculated to require lower energy in each step than the experimental value. Hence, the highest energy in this scenario (not exceeding the experimental value) is that of the transition state **34-Cu**, so that the conversion of **6-Cu** into **36-Cu** can be regarded as the rate-limiting step (RLS) and the reaction should proceed predominantly via an *anti*-addition.[11] Again, one can argue that external attack on an (ethylen)palladium complex, where water has coordinated to Pd to remove negative charge (vide supra), should react faster than the negatively charged complex **6-Cu** (**34-Cu**).

**Scheme 16:**
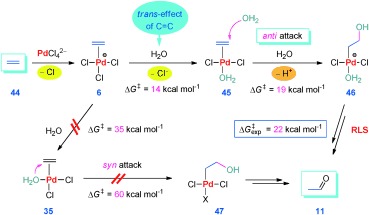
Molecular dynamics calculations including a cubic box of 26 molecules of H_2_O, which simulates the “real” reaction medium and low Cl^−^ concentration.

Recent computational studies, which included aqueous medium, namely a cubic box containing up to 26 molecules of water as a model that is more closely related to the “real” experimental conditions, arrived at the conclusion that *anti*-hydroxypalladation is the favored stereochemical pathway (Scheme 16):[25] First, the initial coordination of tetrachloropalladate to ethylene (**44**) is known to produce the η^2^-complex **6**, which was taken as the starting point. The latter complex should then undergo a ligand exchange if *syn*-migration of the nucleophile from Pd is to be allowed. In view of the geometrical restriction, previous study[11] only considered the *cis*-complex **35**. However, as the new study shows, the calculated activation barrier for its formation from **6** is 35 kcal mol^−1^, which is more than twice as high as that for the pathway leading to its *trans*-isomer **45** (14 kcal mol^−1^). Since the experimental value for the whole Wacker process has been found to be only 22.4 kcal mol^−1^, the formation of **35** appears unlikely. Note that the conversion of **6** into **45** will be assisted by the *trans*-effect (absent in the formation of **35**), which should considerably lower the activation energy, consistent with the calculations. Furthermore, the activation energy required for the *syn*-migration to generate complex **47** from **35** has been calculated to be 60 kcal mol^−1^, far above the experimental value. By contrast, the activation barrier for the *anti*-attack by water on the *trans*-complex **45** to produce **46** was calculated to be only 19 kcal mol^−1^. Hence, it can be clearly seen that none of the activation barriers in the *anti*-pathway (**6** → **45** → **46**) exceeds the experimentally established value[11] for the whole process (22.4 kcal mol^−1^), also indicating that the rate-limiting step should occur after those initial steps, which is consistent with the previous findings.[11] The mechanism in Scheme 16 is also consistent with the rate expression of the Wacker reaction [Eq. (7)], where the rate is inversely dependent on [H^+^] and the square of [Cl^−^]. These advanced calculations thus allow to conclude that the *anti*-hydroxypalladation should be the preferred pathway.[25]

**Scheme 17:**
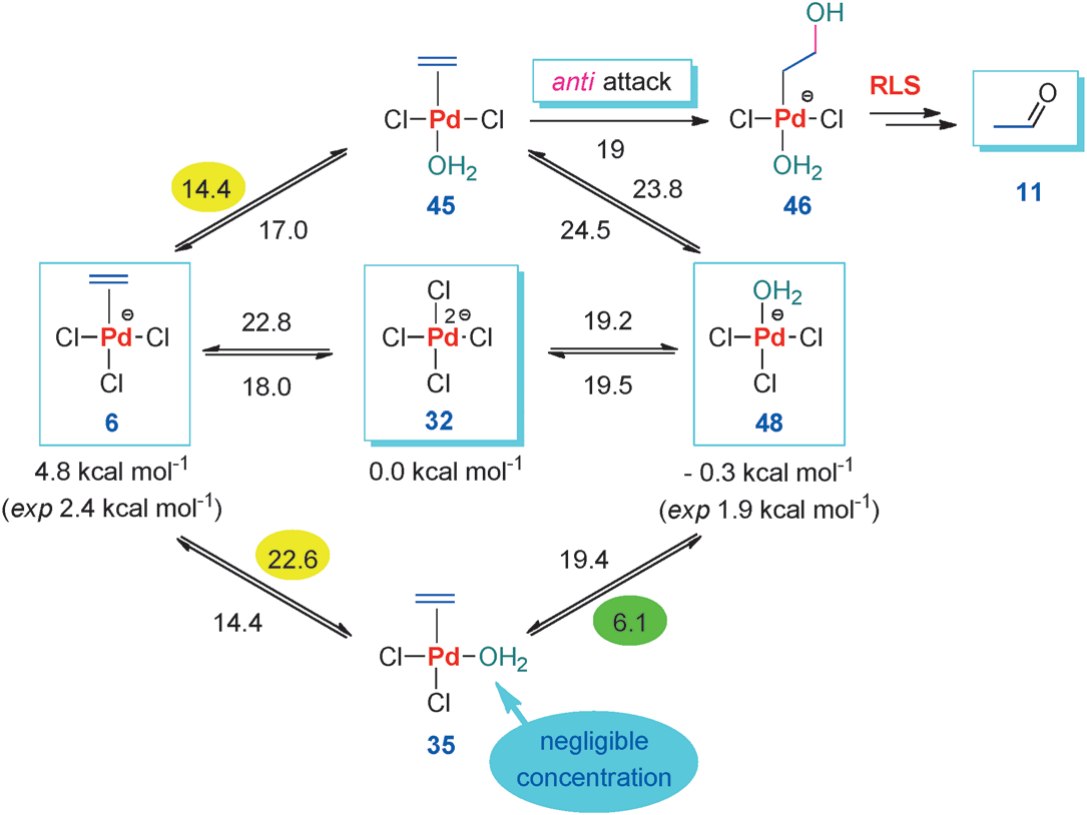
Equilibrium study of ethylene, tetrachloropalladate, and water (Δ*G*^≠^ in kcal mol^−1^).

Another theoretical study, in combination with experimental results (Scheme 17), provides additional support for the *anti*-hydroxypalladation pathway.[26] In the latter study, the equilibria in the system of ethylene, tetracholoropalladate, and water were investigated. Starting with the tetrachloropalladate (**32**), there are two initial reactions to be considered: a ligand exchange with ethylene, generating η^2^-complex **6**, and partial hydrolysis, producing complex **48**. Calculations suggest that the equilibrium should favor **48**, as the energy of its formation is lower than that of **6** (by 5.1 kcal mol^−1^). The experimentally established difference was found to be smaller (0.5 kcal mol^−1^), indicating that both species should be considered for further reactions. Ligand exchange with water in the case of **6** would produce either the *trans*-isomer **45** or its *cis-*counterpart **35**. As discussed in the previous paragraph,[25] conversion of **6** into **45** should be associated with a barrier of 14.4 kcal mol^−1^; the reversed process would require 17.0 kcal mol^−1^, so that this equilibrium should be shifted toward **45**. Formation of the *cis*-complex **35** (from **6**) would require 22.6 kcal mol^−1^, according to these calculations, whereas the reversed reaction can proceed with a barrier of only 14.4 kcal mol^−1^, so that the equilibrium should favor the starting complex **6**. In other words, the displacement of Cl^−^ in **6** by water should preferentially produce the *trans-*complex **45**. The other possible mechanism would be the ligand exchange starting with the aqua-complex **48**. Its reaction with ethylene, generating the *trans*-complex **45** was found to be associated with the activation energy of 23.8 and 24.5 kcal mol^−1^ for the forward and reversed process, respectively. The latter difference is sufficiently small to allow the existence of both species in the equilibrium mixture. A completely different scenario was found for the conversion of **48** into the *cis*-complex **35**. Here, the forward process would require 19.4 kcal mol^−1^, whereas the reversed reaction would be associated with merely a 6.1 kcal mol^−1^ barrier, showing that the equilibrium should be heavily shifted toward **48**. These finding are thus consistent with the previously suggested dominance of the *trans*-complex **45** over its *cis*-isomer **35**. Since the barrier for the *anti*-attack on **45** was previously calculated[25a] to be 19 kcal mol^−1^, whereas the *syn*-migration from **35** would require[25a] 60 kcal mol^−1^, the *syn*-pathway can be ruled out.[26]

**Scheme 18:**
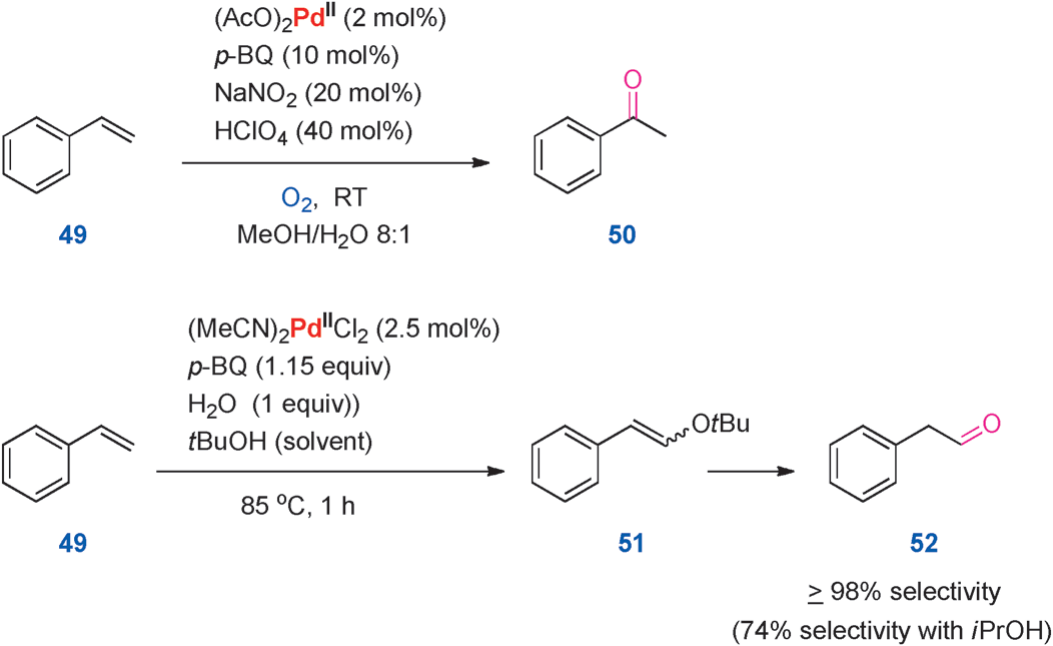
Recent modifications of the Wacker oxidation of terminal olefins and alteration of its regioselectivity.

When extended beyond the original conversion of ethylene to acetaldehyde, the Wacker oxidation has served, with various modifications, as a standard method for oxidation of terminal olefins to produce methyl ketones[27,28] (e.g., **49** → **50** in Scheme 18);[29] this process is referred to as the Wacker–Tsuji oxidation.[27] However, alteration of the regioselectivity in favor of the corresponding aldehydes **52** has recently been achieved simply by using *t*BuOH rather than water. The reaction presumably proceeds via the vinyl ether **51**, resulting from the anti-Markovnikov attack of the bulky nucleophile at the sterically less hindered terminal carbon.[30]

## 3. Amination of Olefins

**Scheme 19:**
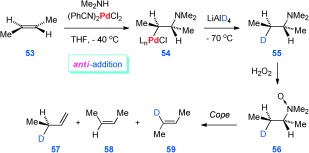
Stereochemistry of the Pd^II^-catalyzed amination of 2-butene.

Related to Wacker oxidation, where the stereochemistry of the initial hydroxypalladation was discussed in detail in the previous chapter, is the amination of olefins. However, in contrast to water and alcohols, amines are not normally considered for analogous Pd-catalyzed reaction, as they are easily oxidized. Nevertheless, a stoichiometric Pd-mediated amination of (*E*)-2-butene (**53**) with dimethyl amine has been successfully investigated at low temperature (Scheme 19).[31] The initially formed aminopalladation product **54** was reduced in situ with LiAlD_4_ (with retention of configuration) to afford the deuteriated amine **55**.[31b] The relative configuration of the latter derivative was established in two steps, involving *N*-oxidation (**55** → **56**) followed by Cope elimination, which is known to proceed with a *syn*-elimination mechanism. The product analysis (**57**–**59**) was consistent with an initial *anti*-addition of Pd^II^ and Me_2_NH across the C=C bond,[31b] in analogy to the mechanism discussed for the Wacker oxidation. Complementary experiments with (*Z*)-2-butene led to the same conclusions.[31,32] Note that before the amine attacks the coordinated olefin, two amine molecules coordinate to palladium. A *syn*-migration of the amine from Pd to the coordinated olefin should be precluded, as this process apparently would be much higher in energy (Figure 1) than the intermolecular *anti*-attack by Me_2_NH, generating **54**. This is in contrast to the Pd-OAc η^3^-species, where the *syn*-delivery via a cyclic transition state is allowed.[33] However, analogous *syn* delivery in the case of the corresponding η^2^-complexes is disfavored (Scheme 13).[22]

## 4. Intramolecular Oxypalladation

**Scheme 20:**
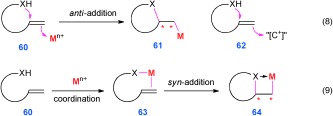
Neighboring group effects in the metal-catalyzed functionalization of olefins.

The stereocontrolled intramolecular nucleophilic attack on a metal–olefin complex (as in **60**), is an important reaction in synthetic organic chemistry,[34] and is analogous to halolactonization, haloetherification, and related reactions employing S, Se, Hg, Tl, Au, and other electrophiles [Scheme 20, Eq. (8)].[1,35] When metals, such as Pd[1,3,5] or Hg,[1,3] are employed as the electrophilic triggers of the reaction, the initially generated organometallic product **61** can be utilized in a subsequent reaction that would allow the construction of a new C=C bond from the C=M bond. The overall result would then be the formation of a C-X and C=C bond, where X is introduced as a nucleophile, and the new C-substituent formally as an electrophile (**62**).[1] Aside from this *anti*-mechanism [Eq. (8)], the *syn*-addition **63** → **64** [Eq. (9)] may also operate (see also Scheme 1), due to the initial coordination (**60** → **63**), known for instance from the vanadium-catalyzed epoxidation.[1c] Examples of both mechanisms and the effects favoring one or the other will be discussed and analyzed in this and subsequent sections.

### 4.1. Intramolecular Oxypalladation Followed by Carbonylation

**Scheme 21:**
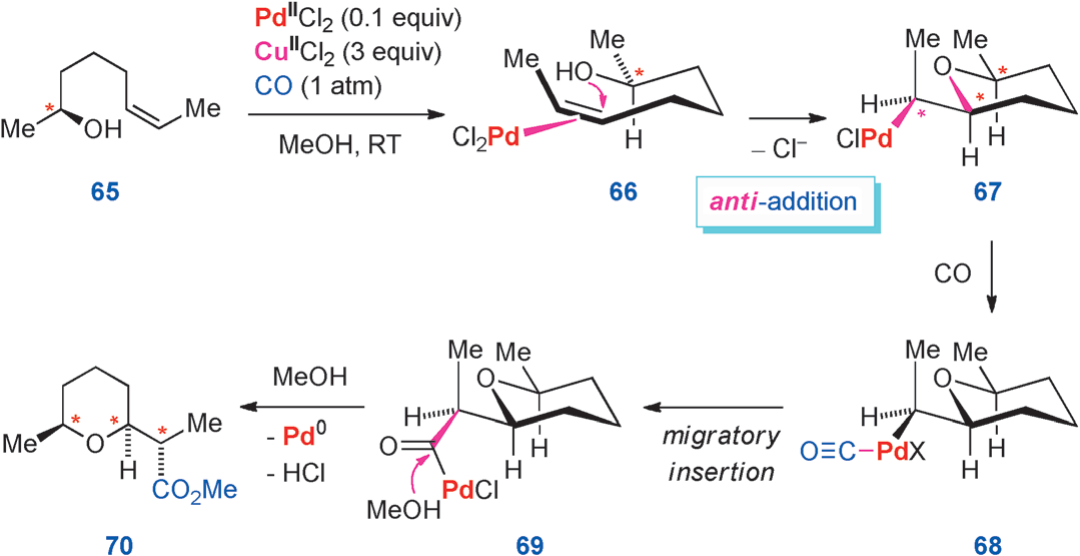
Intramolecular hydroxypalladation controlled by a residing chiral center followed by carbonylation.

The first intramolecular oxypalladation was carried out in conjunction with carbonylation (Scheme 21).[36] Here, the reaction of alkenol **65** with CO and MeOH, catalyzed by Pd^II^, commenced with the η^2^-coordination, followed by an *anti*-attack by the neighboring hydroxyl group. Of the two facial stereoisomers, one is consumed faster (**66**) and generates the tetrahydropyran derivative **67** with high stereocontrol exercised by the original chiral center (note the “equatorial” methyl in **66**). Coordination of carbon monoxide (**68**), followed by migratory insertion then generated complex **69**, which on reaction with methanol produced the desired methyl ester **70** and Pd^0^, whose reoxidation with Cu^II^ completed the catalytic cycle.

**Scheme 22:**
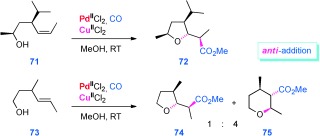
Carbonylative cyclization: the effect of alkene geometry on the regioselectivity.

**Scheme 23:**
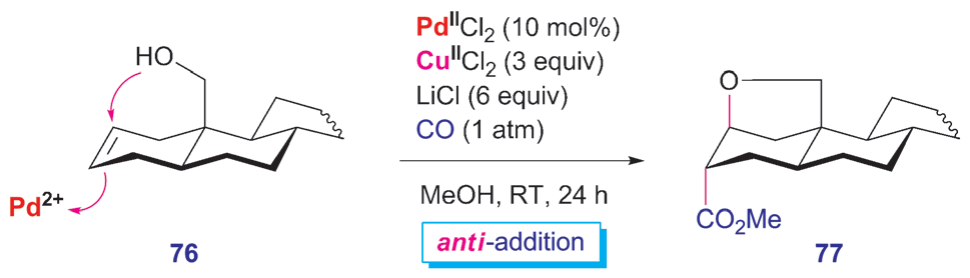
Carbonylative cyclization occuring with pure *anti*-sterechemistry.

Several other examples followed shortly, all featuring the initial *anti*-addition of the electrophilic Pd^II^ and the neighboring OH group across the C=C bond,[37–39] such as the cyclization of **71** and **73** (Scheme 22),[39] and **76** (Scheme 23), followed by carbonylation, as detailed in the previous paragraph.[3a]

**Scheme 24:**
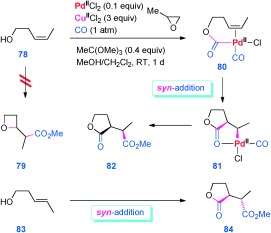
Carbonylative cyclization occuring with pure *syn*-sterechemistry.

All those reactions (Schemes 21—23) occurred with alkenols, whose structures allow 5-*exo*- or 6-*exo*-cyclizations. If the only option is a 4-*exo* process, as in the case of the homoallylic alcohol **78**, the reaction has been found to take a different course (Scheme 24).[40] Here, the C=C bond coordination to Pd^II^ (as described in Scheme 21) becomes unproductive, as cyclization to produce the corresponding oxetane **79** would be too high in energy. Instead, the reaction proceeds through a different channel, namely that involving the initial coordination of CO to Pd, followed by a reaction with the OH group and additional coordination to the C=C bond (**80**). These events are followed by reductive elimination to generate lactone **81** with Pd chelated to the exocyclic carbon in an η^1^-fashion (which formally corresponds to a *syn*-addition across the C=C bond). The cascade is then completed by the second carbonylation to produce **82** (Scheme 24). The *trans*-configured alcohol **83** reacted in the same way to produce the diastereoisomeric lactone **84.** The reaction conditions for these transformations are noteworthy: aside from the standard reoxidation of the resulting Pd^0^ by Cu^II^, propene epoxide was employed to consume the Brønsted acid generated by the reaction, whereas trimethyl orthoacetate was added to secure anhydrous conditions.[40]

Other examples of this reaction course, where the cyclization involving intramolecular oxypalladation is precluded by structural restrictions in the homoallylic arrangement, have been reported but without addressing the stereochemistry issues.[41]

### 4.2. Intramolecular Oxypalladation Followed by β-Hydride Elimination

**Scheme 25:**
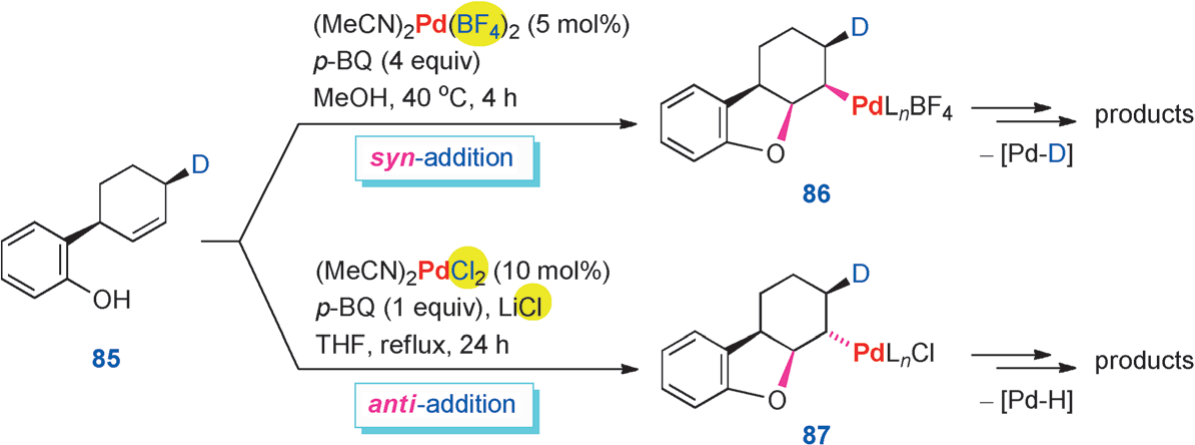
Switching between *syn*- and *anti*-mechanism in the intramolecular hydroxypalladation as a function of the anion coordinated to Pd.

The stereochemistry of the Pd^II^-catalyzed ring closure of alkenols has been investigated with the aid of the stereospecifically deuteriated substrate **85**, using *p*-benzoquinone (*p*-BQ) as the terminal oxidant (Scheme 25).[42] Since intermediates in a catalytic reaction are difficult to isolate, the steric course was inferred from the products of the subsequent β-H elimination, which is known to occur as a *syn*-process. With the weakly coordinating BF_4_^−^ anion at Pd^II^, the free phenolic hydroxyl group tends to coordinate to the metal [as in Eq. (9), Scheme 20], which leads to a π-olefin complex where Pd is bound to the top face of cyclohexene. A *syn*-oxypalladation then produces **86**, which on subsequent β-hydride elimination removed the *cis*-positioned deuterium. By contrast, coordination to the neighboring hydroxyl is precluded in the case the strongly coordinating Cl^−^ at Pd^II^ (especially when additional LiCl is present) and the reaction thus proceeded with *anti-*stereochemistry to give **87**, as revealed by the β-H elimination of the *cis*-disposed proton.[33,42,43]

**Scheme 26:**
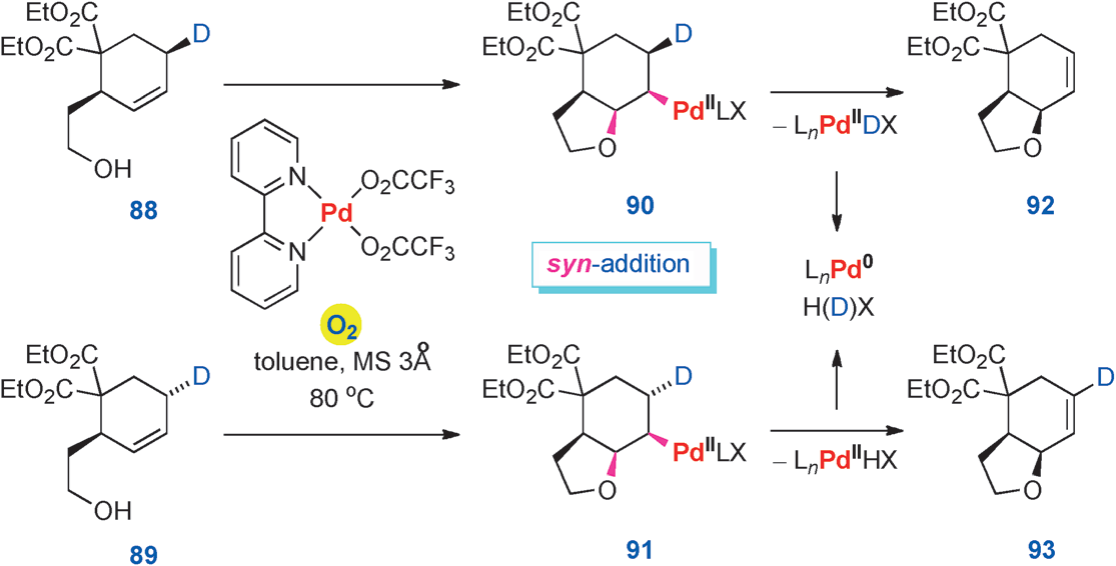
*syn*-Mechanism in the intramolecular hydroxypalladation.

The latter study used phenolic hydroxyl as a neighboring group, whose coordination capability can differ from that of an ordinary alcohol due to the difference in the p*K*_a_. Therefore, two diastereoisomerically deuteriated substrates **88** and **89** were investigated by Stoltz and co-workers (Scheme 26).[44] The catalyst employed possessed the weakly coordinating trifluoroacetate anions and the reaction was found, in both cases, to follow the *syn*-pathway, as revealed by the β-hydride elimination of **90** and **91** to give **92** and **93**, respectively. These results are in full agreement with the first reaction shown in Scheme 25. Notably, the catalyst in Scheme 26 allowed the use of molecular oxygen as the stoichiometric oxidant, avoiding the requirement to employ a mediator, such as Cu^II^ or quinone/metal macrocycle.[44] The use of the bipyridine ligand presumably keeps the reduced palladium in solution and gives it time to be reoxidized. This study clearly demonstrates that for intramolecular alkoxypalladation, coordination of the neighboring hydroxy group is a powerful process that favors *syn*-addition. When the alkoxy group is first coordinated to palladium in the π-olefin complex of **88** and **89**, there will be no competing pathway via *anti*-attack.

Solvents (e.g., MeOH vs MeCN) have been found to have an effect on the regiochemistry of the β-hydride elimination but that study did not address the stereochemical issues.[45]

### 4.3. Intramolecular Oxypalladation Followed by Heck Addition

**Scheme 27:**
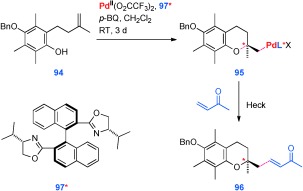
Pd^II^-Catalyzed ring closure followed by Heck addition.

Phenolic olefin **94** has been reported to undergo a Pd-catalyzed cyclization (with *p*-BQ as the terminal oxidant) in the presence of Michael acceptors, such as methyl vinyl ketone, to produce **96** with high enantioselectivity, owing to the chiral ligand **97** (Scheme 27). Although the steric course of the initial step (**94** → **95**) has not been investigated, it can be assumed to proceed via a *syn*-mechanism, owing to the weakly coordinating anion (CF_3_CO_2_^−^) and the presence of **97**, whose ligating properties are likely to mirror those of the bipyridine, featured in Scheme 26.[46]

### 4.4. Intramolecular Oxypalladation Followed by Arylation

**Scheme 28:**
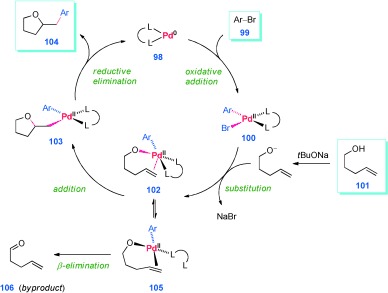
Pd^0^-Catalyzed ring closure followed by arylation.

The Pd^II^ species **100**, generated in the catalytic cycle from the Pd^0^ complex **98** on an oxidative addition to aryl halide **99**, has been shown to react with the alkoxide generated from the bishomoallylic alcohol **101**, giving rise to the cyclization product **104** (Scheme 28).[47] The corresponding alkenyl aryl ether that would be normally expected for these conditions (typical for the Hartwig–Buchwald coupling) has not been observed. The reaction has been suggested to proceed via the η^2^-complex **102** (with pentacoordinated Pd), predisposed to the *syn*-cyclopalladation, generating the tetrahydrofuran intermediate **103**. The latter species then undergoes reductive elimination to afford **104** as the final arylated product, thereby regenerating Pd^0^ **98** for the next catalytic cycle. Aldehyde **106** has been isolated as a byproduct,[47] apparently arising from **105** via a β-hydride elimination.

**Scheme 29:**
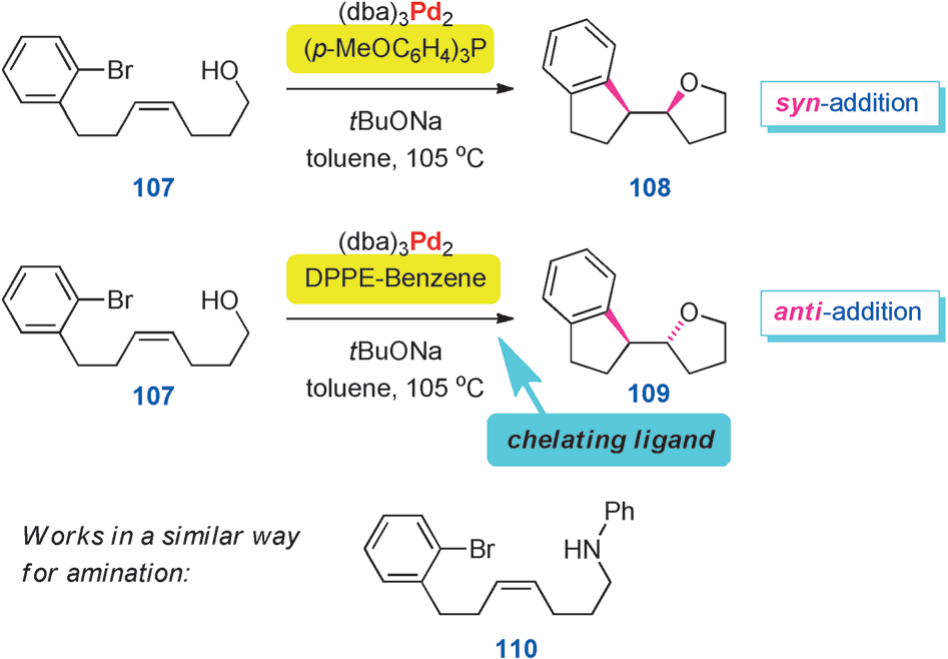
Stereochemistry variation in the Pd^0^-catalyzed ring closure followed by arylation.

The stereochemistry of the latter reaction was investigated with the aid of the *cis*-alkenol **107** (Scheme 29).[48] As expected (based on the discussion in the previous paragraph), the reaction proceeded as a *syn*-addition to produce **108**. However, by using DPPE as a strongly chelating ligand, which apparently prevents the alkoxide coordination to palladium, the mechanism was pushed toward *anti*-addition, giving rise to the diastereoisomeric product **109**. Amine **110** reacted in the same way.[48]

**Scheme 30:**
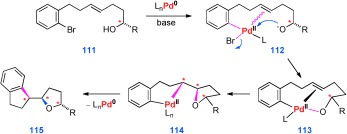
Stereocontrol of the cyclization by a residing chiral center.

The *syn*-addition mechanism has been masterly utilized in the cyclization, where the stereochemistry was controlled by the residing chiral center in the secondary alcohol **111** (Scheme 30).[48] Here, the initial oxidative addition to a Pd^0^ catalyst produces **112**, where the C=C bond is coordinated to Pd. Two diastereofacial ways of this coordination can be considered, so that a mixture might be expected. However, the subsequent replacement of bromide in the Pd coordination sphere with the neighboring alkoxy group (to generate **113**) is apparently faster for one stereoisomer (as, e.g., in Scheme 21), which in view of the reversibility of the η^2^-coordination results in dynamic stereodifferentiation.[49] Hence, isomer **114** is thus formed preferentially and the subsequent reductive elimination affords the cyclization product **115** of high diastereopurity.[48]

## 5. Intramolecular Amidopalladation

**Scheme 31:**
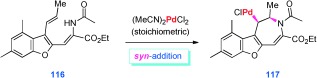
Ring-closing amidopalladation occuring with *syn*-stereochemistry.

The first amidopalladation, analogous to alkoxypalladation, was explored in a stoichiometric experiment with enamide **116** and found to produce the azepine derivative **117** with the new C=N and C=Pd bonds related in a *cis*-fashion, which correspond to *syn*-addition (Scheme 31).[50]

### 5.1. Intramolecular Amidopalladation Followed by β-Hydride Elimination

**Scheme 32:**
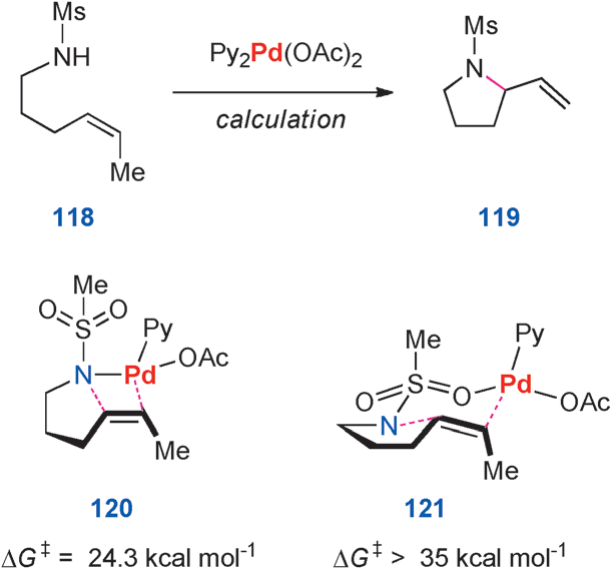
Computational analysis of ring-closing amidopalladation proceeding with *syn*-sterechemistry.

Assuming a similar mechanism for the Pd^II^-catalyzed cyclization of the alkenyl sulfonamide **118** (Scheme 32), two transition states **120** and **121**, differing in the mode of Pd coordination can be proposed. The former species (with Pd coordinated to the nitrogen) was found to be much lower in energy than the latter by quantum chemistry calculations.[51,52]

**Scheme 33:**
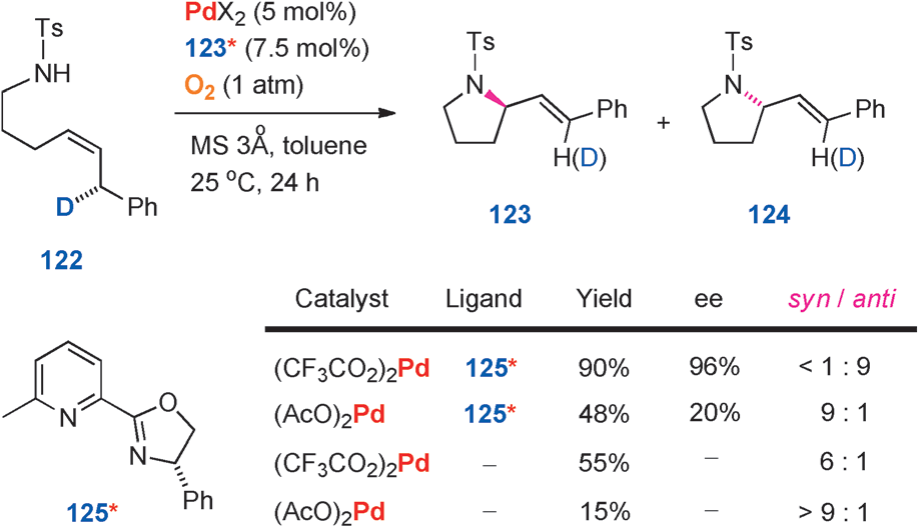
Anion effect on the stereochemistry of the sulfonamidopalladation.

Further investigation revealed that the stereochemistry of amidopalladation is subjected to delicate effects of the anion in the original PdX_2_ catalyst and ligand (Scheme 33).[52,53] With the monodeuteriated substrate **122**, the cyclization was catalyzed by two Pd^II^ salts in the presence or absence of ligand **125** and the products were analyzed for the content of deuterium in the resulting cyclic olefins **123** and **124**. The latter analysis demonstrated that the chelation of Pd (and thus the *syn*-pathway) is suppressed when the combination of (CF_3_CO_2_)_2_Pd and ligand **125** is used, whereas the remaining reactions were dominated by the *syn*-pathway.[53b]

**Scheme 34:**
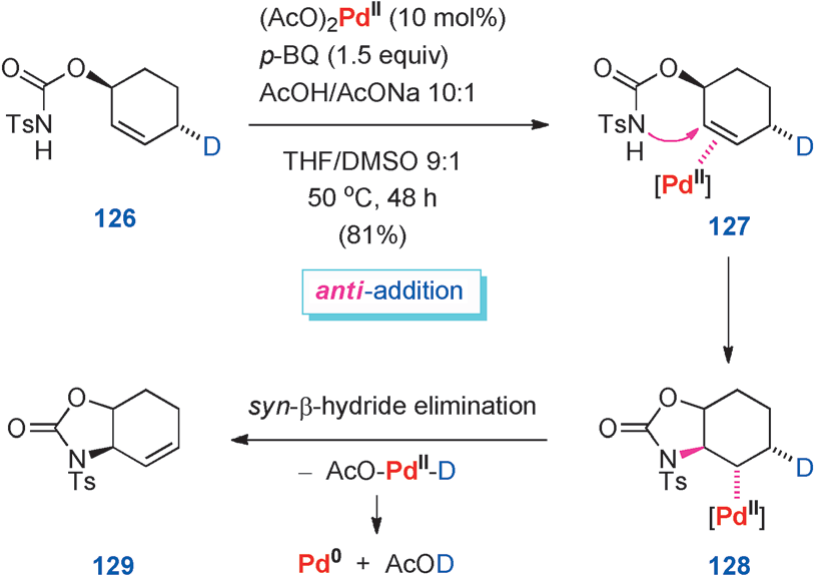
Stereochemistry of sulfonamidopalladation with cyclic substrates.

To add to the complexity of the mosaic, the cyclic, stereospecifically deuteriated sulfonamide **126** was found to be cyclized via the *anti*-pathway (with **127** and **128** as intermediates), giving rise to **129**, which lacks the label (Scheme 34).[54] In this instance the reaction was catalyzed by Pd(OAc)_2_ and would be expected to occur via the *syn*-addition pathway according to Scheme 33. However, in the previous study the final oxidant was molecular oxygen, whereas in Scheme 34 it was *p*-BQ, which is known to coordinate to Pd in an η^2^-intermediates[55] and this should be regarded as a contributing factor. Also, the fact that the reaction was run under slightly acidic conditions may inhibit coordination of the tosylamide. Note that a stoichiometric experiment with the cyclopentene analogue of **126**, *N*-coordinated to (*t*Bu_2_bipy)PdCl, has been found to cyclize via the *syn*-pathway in DMSO in the presence of molecular oxygen (to simulate the catalytic conditions).[53a] Clearly, more studies are required to define the relationship between the reaction conditions and the mechanism.

**Scheme 35:**
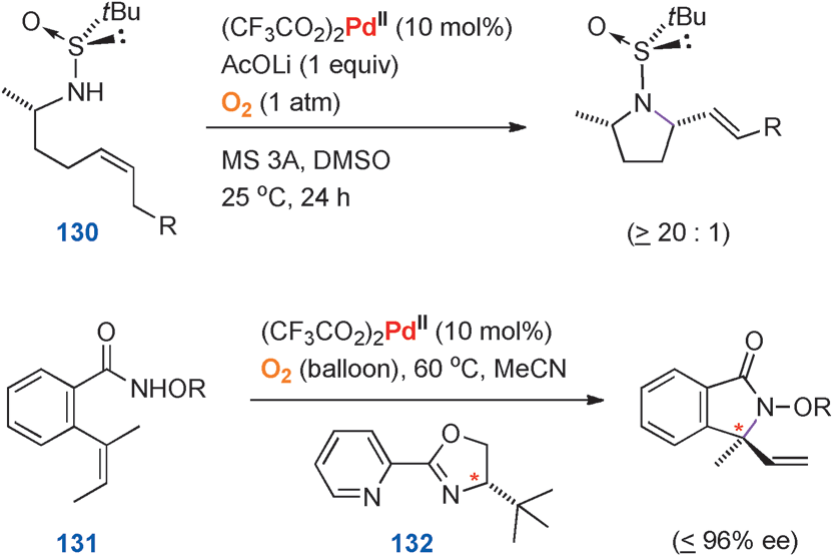
Asymmetric amidopalladation.

The *syn*-mechanism has been assumed (but not proven) for the cyclization of **130**[56] and **131**[57] (Scheme 35), where the chiral control is exercised by a chiral sulfinimide group and chiral ligand **132**, respectively.

**Scheme 36:**
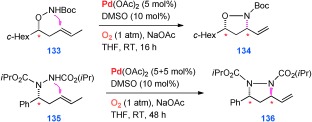
Diastereoselective 1,3-amidation.

Two other amidation reactions have been reported recently (Scheme 36) but their stereochemistry has not yet been elucidated.[58] In accord with the previous observations (vide supra), the hydroxylamine derivative **133** afforded the *cis*-configured isoxazolidine **134** (≥30:1 dr), whereas its hydrazine analogue **135** produced the *trans*-configured pyrrazolidine derivative **136** (≥30:1 dr).[58a] The latter products can be regarded as surrogates of 1,3-amino alcohols and diamines, respectively.

**Scheme 37:**
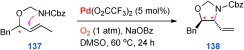
Diastereoselective 1,2-amidation.

Finally, the *O*-allyl hemiaminal **137**, prepared from the corresponding allylic alcohol and AcOCH_2_NHCbz, has been shown to undergo an analogous cyclization, giving rise to the *trans-*configured oxazolidine **138** (Scheme 37) but again without specification as to the actual stereochemistry of the cyclopalladation step.[59] This strategy has been developed as an approach to 1,2-*syn*-amino alcohols and employed in the synthesis of acosamine.[59]

### 5.2. Intramolecular Amidopalladation Followed by Arylation

**Scheme 38:**
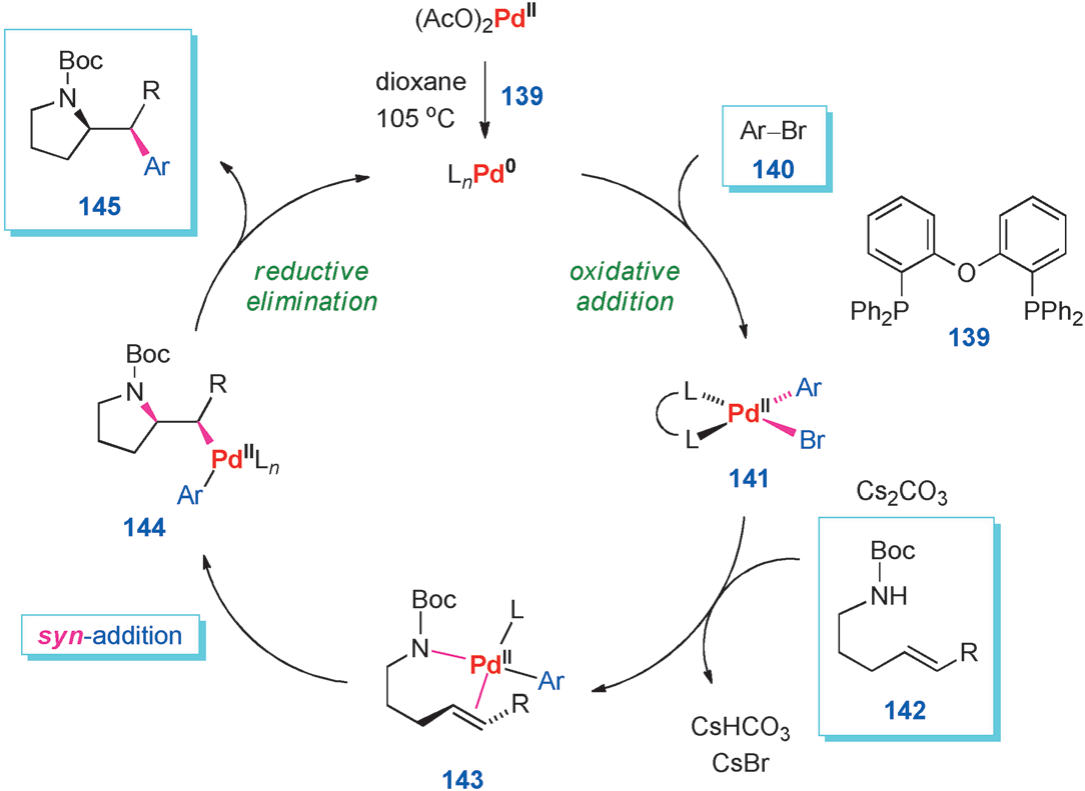
Intramolecular amidopalladation combined with arylation.

The cascade of catalytic amidopalladation of olefins and arylation has been developed in parallel with oxypalladation (cf. Section 4.4). Thus, the Pd^0^ species, generated from Pd(OAc)_2_ and the diphosphine ligand **139** (Scheme 38),[60] has been shown to catalyze the cyclization of the Boc-functionalized aminoalkene **142** to produce the piperidine derivative **145** with high diastereoselectvity. The latter outcome has been rationalized in a similar way as in the case of oxypalladation, namely by the initial oxidative addition to generate the Pd^II^ complex **141**, whose reaction with **142** (upon deprotonation with Cs_2_CO_3_) generates the Pd=N complex **143**, where Pd is also coordinated to the C=C bond. The key addition across the C=C bond thus occurs with a *syn*-mechanism to generate the η^1^-complex **144**, which affords the final product **145** by reductive elimination and Pd^0^ that enters the next catalytic cycle.[60,61]

**Scheme 39:**
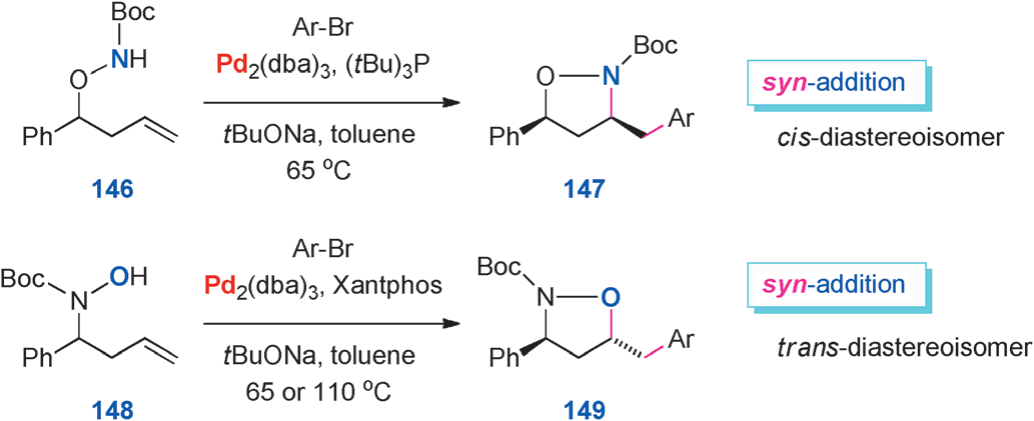
Intramolecular amidopalladation/arylation of hydroxylamine derivatives.

**Scheme 40:**
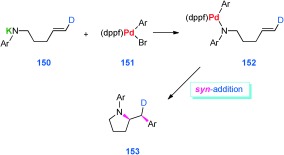
Mechanism of the stoichiometric aminopalladatio/arylation elucidated by isotopic labeling (Ar=*p*-CF_3_C_6_H_4_).

The cyclization of the isomeric derivatives of hydroxylamine **146** and **148** (Scheme 39) was expected to proceed via the *syn*-mechanism[62] in analogy to the related cyclizations highlighted in Scheme 38 (see also the evidence presented in Scheme 40). The *syn*-mechanism was than proved by isotopic labeling at the terminus of the double bond. Note, however, that while **146** gave the *cis*-disubstituted isoxazolidine **147** (typically with ≥20:1 dr), its positional isomer **148** that reacts by forming the C=O (rather than C=N) bond, furnished mainly the *trans*-product **149** (7:1 dr). This change of stereochemistry was attributed to the steric interference by the Boc group 1,2-related to the phenyl in **148**; a transition state leading to **149** is believed to be lower in energy than that producing the *cis*-diastereoisomer (both using the *syn*-addition mechanism).[62]

Stoichiometric experiments (Scheme 40),[63] employing the potassium salt **150**, stereospecifically deuteriated at the terminus of the double bond, and the ArPdBr complex **151**, lend further credence to the *syn*-mechanism (via **152**).[63] The same conclusion has been arrived at for an intermolecular, stoichiometric amination of (*Z*)-CHD=CHD with Ph_2_N-[Pd][64] and for an intramolecular amidation of a deuterated cyclopentene substrate followed by β-H elimination.[53a]

**Scheme 41:**
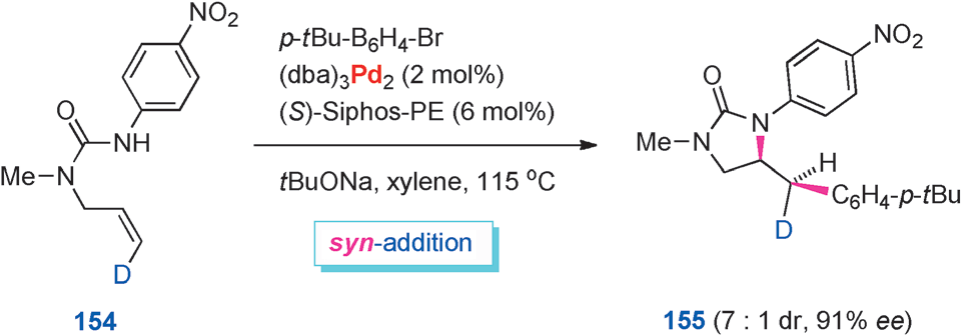
Asymmetric aminopalladatio/arylation.

Another piece of complementary evidence was obtained from the catalytic cyclization of the deuteriated urea derivative **154**. The relative configuration of the major product **155** was found to be consistent with the *syn*-mechanism of the addition (Scheme 41).[65]

**Scheme 42:**
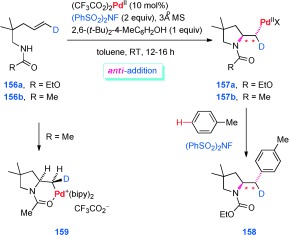
Intermolecular amidopalladation followed arylation with C=H activation.

On the other hand, cyclization of the deuteriated acetamide **156 a**, carried out in the absence of a strong base, afforded the arylated pyrrolidine derivative **158** (Scheme 42) as a result of an *anti-*amidopalladation (**156 a → 157 a**). The Pd^II^ species **157 a** thus generated is oxidized with *N*-fluorosulfonimide to afford the corresponding Pd^IV^ complex. The latter intermediate then effects an electrophilic attack on toluene with retention of configuration to produce the C-arylated derivative **158** (thus accomplishing a C=H activation). The stereochemistry of the *anti*-aminopalladation has been confirmed by a stoichiometric experiment, in which **156 b** was converted into the stable bipy complex **159**, characterized by NMR spectroscopy.[66] The dramatic difference in the two mechanisms described in Schemes 41 and 42 apparently originates in the actual reaction conditions: in the former case, the amide-type nitrogen in **154** is deprotonated by a strong base, which increases its propensity to coordinate the palladium catalyst. In the latter case the base is absent and the palladium apparently prefers to first coordinate to the C=C bond; the resulting species then undergoes a traditional attack from the opposite face as in any classical electrophilic addition.

Numerous other examples highlight the application of this palladium-catalyzed intramolecular amidoarylation of alkenes in the synthesis of various nitrogen heterocycles.[67,68] In the most recent examples both amidation and arylation were carried out as intramolecular processes to produce polycyclic structures.[69] This area has also been reviewed.[70]

### 5.3. Intramolecular Amidopalladation Followed by a Second Amidation

**Scheme 43:**
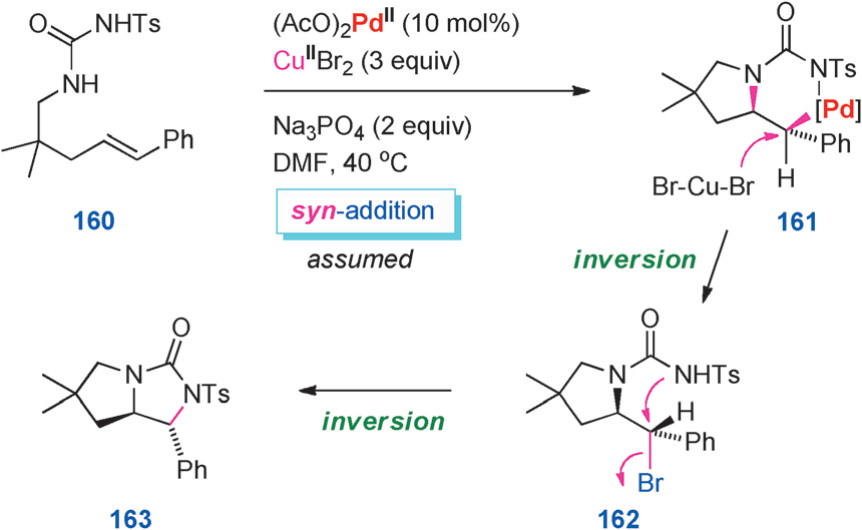
Catalytic intramolecular bis-amidation.

The amine-type nitrogen, being trivalent, offers another dimension in synthetic strategy that is not available to the divalent oxygen: while intramolecular alkoxypalladation produces a cyclic ether that cannot be further elaborated on the oxygen, the analogous amidopalladation features an additional *N*-substituent that can be involved in the subsequent events. Thus, the urea derivative **160** has been shown to undergo double cyclization to give the *trans-*configured bicyclic product **163** (Scheme 43).[71] The stereochemistry of this cascade was rationalized as follows: the initial amidopalladation (presumably proceeding with the established *syn*-mechanism) generates the Pd-chelate **161**, in which Pd is replaced by bromide from CuBr_2_ with S_N_2 inversion and the resulting bromo derivative **162** undergoes cyclization with a second inversion to produce **163**.[71] However, an alternative pathway, involving *anti*-amidopalladation, followed by oxidative cleavage of the C=Pd bond by S_N_ 2 displacement with the nitrogen, cannot be excluded.

**Scheme 44:**
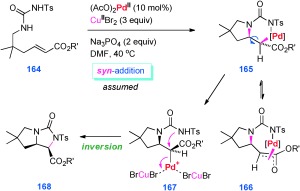
Catalytic intramolecular bis-amidation.

The related urea derivative **164** (with an ester group in the place of a phenyl of the previous example), also underwent a successful cyclization (Scheme 44) but producing the *cis*-derivative **168** (in contrast to the *trans*-isomer resulting from the previous example). The discrepancy was reconciled by assuming just one inversion: here, complex **165** (analogous to **161**), instead of undergoing the Br^−^ initiated inversion, is believed to coordinate CuBr_2_ (**167**), and Pd itself then serves as a leaving group in the ring closure,[72] so that **168** is formed with a single inversion. The difference between the reactivities of **161** and **165** has been attributed to the enolate-type equilibrium **165** ⇄ **166** that is not available to **161**.[71] Nevertheless, an alternative *anti*-amidopalladation, followed by an oxidative cleavage of the C-Pd by CuBr_2_ with inversion, can also be considered.

**Scheme 45:**
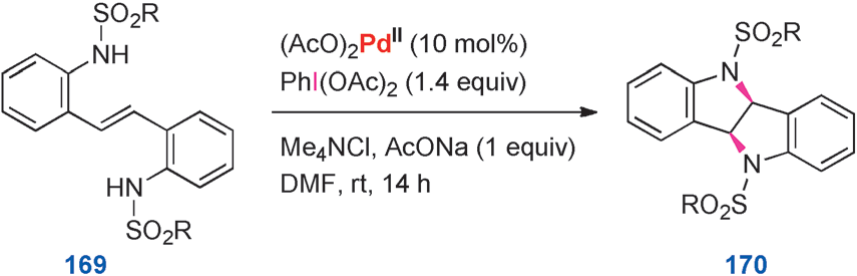
Catalytic intramolecular double-amidation.

A different type of bis-amidation has been reported for the stilbene-derived bis-sulfonamide **169** (Scheme 45).[73] Here, the first cyclization presumably proceeds with *anti*-stereochemistry[74] and the arising Pd^II^ σ-complex is believed to be oxidized by PhI(OAc)_2_ to generate Pd^IV^, which enables its replacement with the second sulfonamide group to produce **170**.[73]

**Scheme 46:**
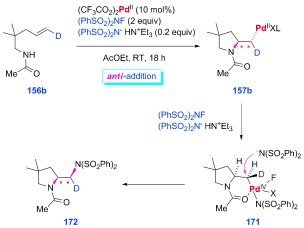
Intermolecular amidopalladation followed another amidation.

The deuteriated substrate **156 b** was used again to elucidate the diamidation process that afforded the pyrrolidine derivative **172** (Scheme 46). The latter outcome corresponds to the initial *anti* amidopalladation generating the Pd^II^ species **157 b** (as in Scheme 42). The latter complex then undergoes oxidation with (PhSO_2_)_2_NF, giving rise to the Pd^IV^ species **171**, which is then converted into the final product **172** on reaction with the imide anion via an S_N_2 inversion.[66]

### 5.4. Intramolecular Amidopalladation Followed by Carbonylation

**Scheme 47:**
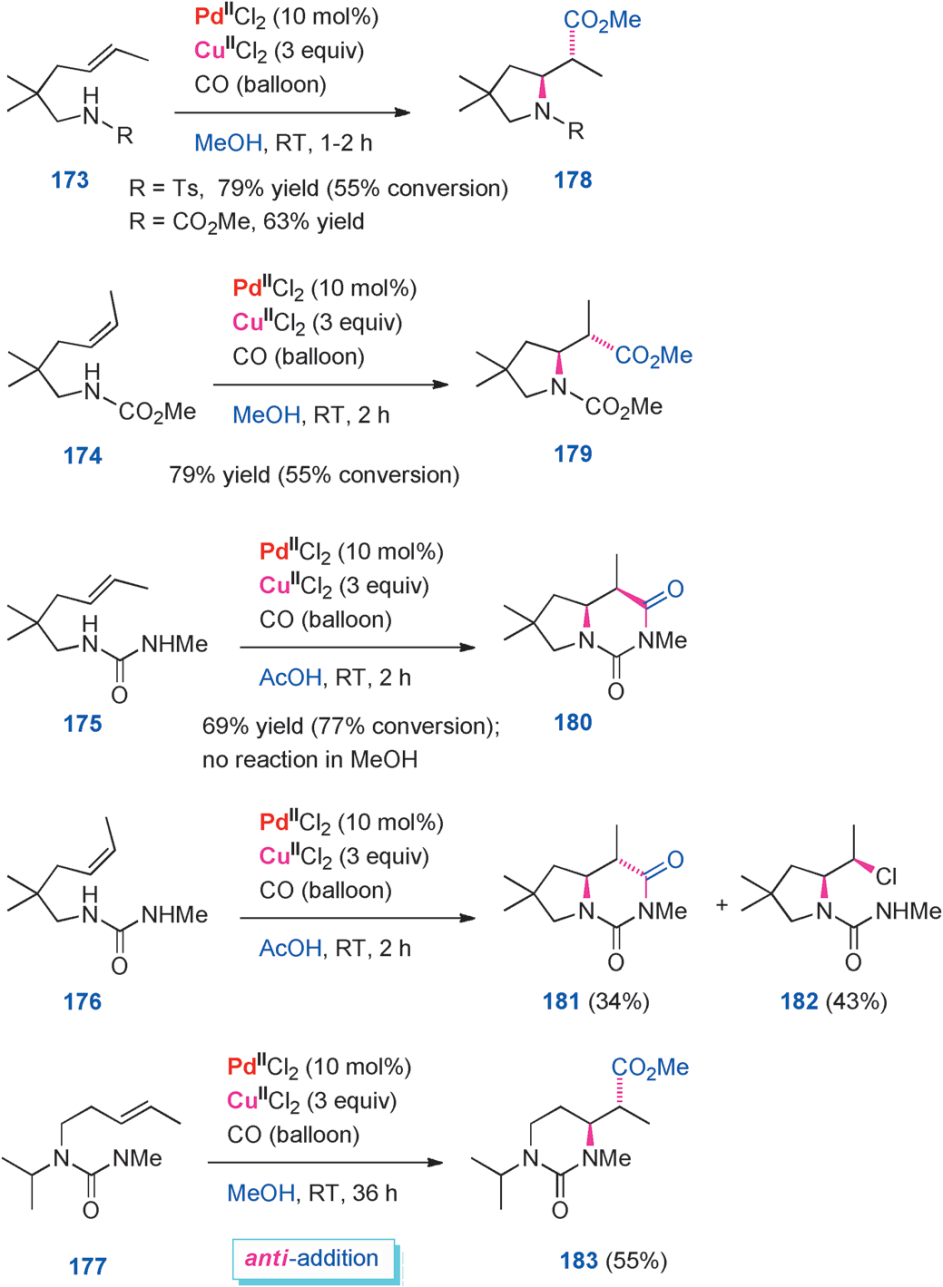
Amidopalladation followed by carbonylation.

Intramolecular amidopalladation of olefinic amides, sulfonamides, and ureas **173**–**177** in the presence of carbon monoxide and methanol (Scheme 47)[75] has been reported to afford products corresponding to the *anti*-mechanism, irrespective of the configuration of the C=C bond in the starting molecule. The reaction conditions are mild: atmospheric pressure of CO, room temperature, and CuCl_2_ as the terminal oxidant. Both 5-*exo* and 6-*exo* cyclizations were attained. In the case of the urea derivatives **175** and **176**, trapping of the acyl–Pd intermediate with the second nitrogen was observed in the absence of methanol to give **180** and **181**. In the latter instance, the chloro derivative **182** was also obtained, apparently as a product of the S_N_2 substitution of Pd in the intermediate by chloride ion (as in the case of **21** and **161**).[75]

**Scheme 48:**
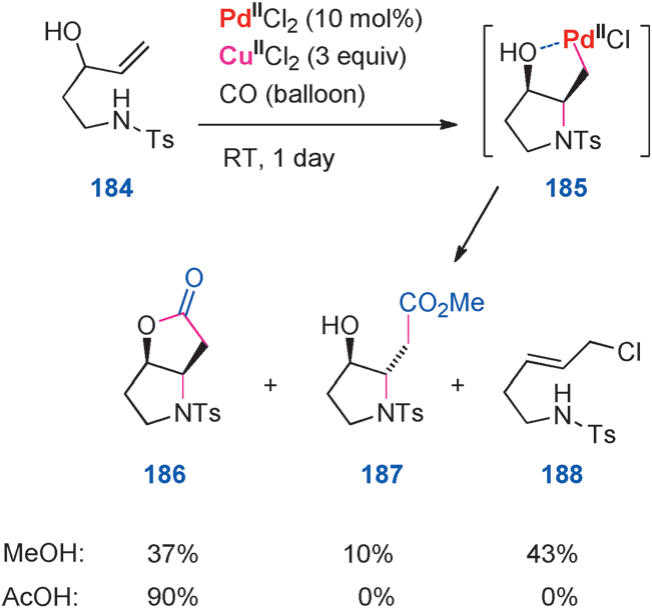
Amidopalladation followed by lactonization.

With a free hydroxy group in the molecule as in **184**, the initial amidation has been found to continue by lactonization to the neighboring hydroxyl (Scheme 48).[76] The yield of the resulting lactone **186** was maximized by replacing MeOH (as a solvent and competitor) with AcOH. While the stereochemistry of the initial amidopalladation has not been investigated in this instance, it was assumed to correspond to the *anti*-delivery. The diastereoselectivity is controlled by the hydroxy group, presumably by Pd coordination (**185**).

**Scheme 49:**
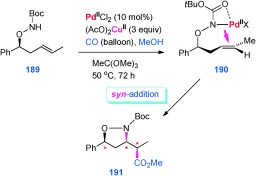
Amidopalladation of hydroxylamine derivatives followed by carbonylation.

Finally, the Boc-protected hydroxylamine derivative **189** has been shown to undergo the cyclization/carbonylation with an overall *syn*-addition across the double bond, giving rise to the isoxazoline derivative **191** (Scheme 49).[77] The stereochemical outcome[78] apparently originates from the initial coordination of the Boc group to Pd^II^ (**190**), followed by *syn*-amidopalladation and subsequent carbon monoxide cleavage of the Pd=carbon bond with retention of configuration. The striking contrast between the stereochemistry of the carbonylative cyclization of the amides shown in Scheme 47 and the latter case is puzzling, as the reaction conditions are very similar. One explanation is that CuCl_2_ was used in Scheme 47, whereas Cu(OAc)_2_ was employed in the cyclization of **189** (Scheme 49). The use of CuCl_2_ leads to a high chloride concentration, which makes it more difficult for the amide to coordinate to palladium, and hence the *anti*-pathway should be favored. Furthermore, the latter reaction was carried out in the presence MeC(OMe)_3_, which could modify the pH of the mixture and thus improve the propensity of the ONHCO_2_(*t*Bu) group to coordinate to palladium.

## 6. Intermolecular Amidopalladation

The intramolecular amidopalladation has been shown to mostly proceed as a *syn*-addition, which apparently stems from the considerable entropic advantage of the neighboring group over an external nucleophile. This factor is absent in intermolecular additions, which may change the stereochemistry, as demonstrated convincingly for the Wacker oxidation (see Chapter 2).

### 6.1. Amidoacetoxylation

**Scheme 50:**
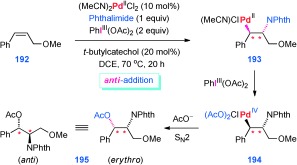
Intermolecular amidoacetoxylation of internal olefins.

While terminal olefins have been shown to readily undergo intermolecular Pd-catalyzed amidoacetoxylation,[79] their internal counterparts resisted a number of attempts. Finally, *cis*-olefins, such as **192** (Scheme 50), have been successfully converted into the amidoacetoxylation products on reaction with phthalimide in the presence of PhI(OAc)_2_ as the oxidizing agent of palladium. The reaction was originally formulated as proceeding via an initial *syn*-amidopalladation. However, recent re-investigation,[80] prompted by the results of the related diamidation (see the next subchapter), demonstrated that the original structural assignment of the product[81] was incorrect. This finding led to a revision of the mechanism, according to which the initial amidopalladation of **192**, catalyzed by Pd^II^, proceeds as a pure *anti* process (rather than *syn*) to generate the palladium(II) intermediate **193** that is subsequently oxidized by the hypervalent iodine reagent to produce the Pd^IV^ species **194**. The latter reaction prevents the usual β-elimination and is followed by the S_N_2 replacement of palladium with acetate (i.e., with inversion of configuration[82]) to afford the final product **195** that is *anti*-configured (rather than *syn*), according to X-ray analysis.[80,83] Noteworthy is also the high regioselectivity of this cascade, owing to the preferential attaching of the palladium moiety to the benzylic position (**193**).[84]

**Scheme 51:**
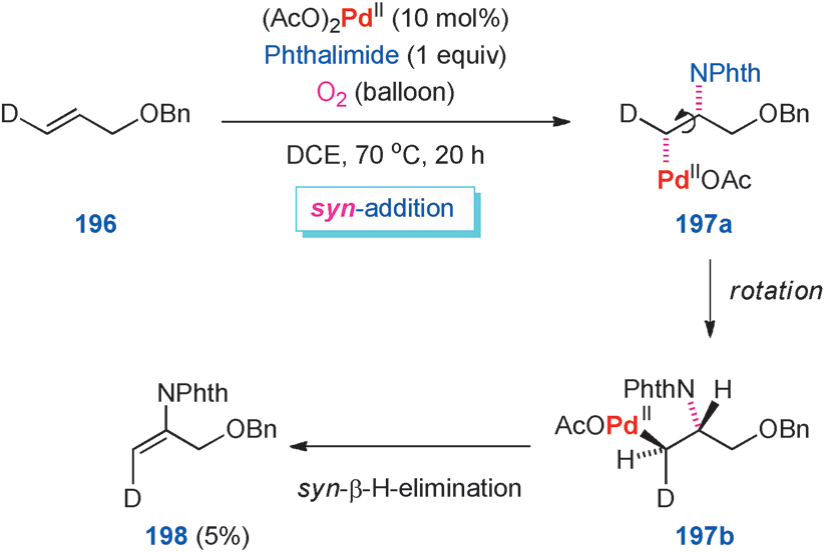
Intermolecular aerobic amidoacetoxylation of terminnal olefins.

By contrast, the behavior of terminal olefins has been shown to be more complicated: Thus, with the aid of the stereospecifically deuteriated olefin **196** (Scheme 51), the reaction was found not to be stereoselective, giving rise to a ∼4:3 mixture of diastereoisomeric amidopalladation products (analogous to **195**). The latter outcome has been attributed to the poor stereoselectivity of the replacement of Pd with acetate in the final step. On the other hand, under aerobic conditions (i.e., in the absence of the hypervalent iodine reagent), the formation of the (*E*)-enamide **198** was ascribed to the *syn*-addition, generating **197**, followed by the ordinary *syn*-stereoselective elimination of [PdH]. However, it is pertinent to note that the product **198** was isolated in merely 5 % yield (together with 70 % of the recovered starting material).[80]

### 6.2. Diamidation

**Scheme 52:**
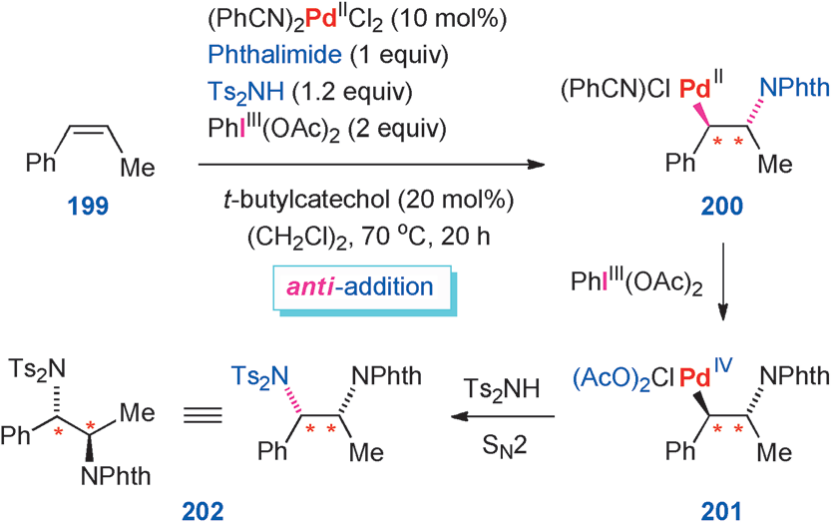
Intermolecular diamidation of internal olefins.

In analogy to acetoxyamidation, the recently developed diamidation (Scheme 52) has also been found to be specific to *cis*-olefins, such as **199**. The formation of the *anti-*configured of final product **202** has been rationalized by the initial *anti-*addition of Pd^II^ and phthalimide to generate **200**, which is then oxidized with the hypervalent iodine reagent to produce the Pd^IV^ intermediate **201**. The final displacement of Pd with ditosyl imide is believed to proceed with inversion (as in the previous case[80,82]) to produce the vicinal diamido derivative **202**.[85]

## 7. Overview of the Stereochemical Outcome in Nucleophilic Addition

The *syn*/*anti*-dichotomy in the nucleophilic additions was discussed mainly for oxygen and nitrogen nucleophiles. The stereochemical trends are summarized in Table 1, which covers cyclization processes (entries 1–28) and intermolecular reactions (entries 29–33).

**Table 1:** Stereochemistry of the Pd^II^-catalyzed addition of nucleophiles to C=C bond: intramolecular (entries 1–28) and intermolecular (entries 29–33).

Entry	Reaction[a]	Scheme	*syn*/*anti*	PdX_2_	Ligand	Oxidant	Solvent	Additive	*T* [°C]	Ref.
1	***A***[b]	21	*anti*	PdCl_2_	–	CuCl_2_	MeOH	CO	RT	[36]
2	A[b]	22	*anti*	PdCl2	–	CuCl2	MeOH	CO	RT	[39]
3	***A***[b]	23	*anti*	PdCl_2_	–	CuCl_2_	MeOH	CO, LiCl	RT	[3a]
4	***A***^[b,c]^	24	*syn*	PdCl_2_	–	CuCl_2_	MeOH, CH_2_Cl_2_	CO, MeC(OMe)_3_	RT	[40]
5	***B***[b]	25	*anti*	PdCl_2_	MeCN	*p*-BQ	THF	LiCl	reflux	[42]
6	***B***[b]	25	*syn*	Pd(BF_4_)_2_	MeCN	*p*-BQ	MeOH	–	40	[42]
7	***B***[b]	26	*syn*	Pd(O_2_CCF_3_)_2_	bipy[h]	O_2_	toluene	3 Å MS	80	[44]
8	***C***[b]	28	*syn*	Pd_2_(dba)_3_	dpe-phos (**139**)[h]	–	THF	*t*BuONa, ArBr	65	[47]
9	***C***[b]	29	*syn*	Pd_2_(dba)_3_	(4-MeOC_6_H_4_)_3_P	–	toluene	*t*BuONa, ArBr[l]	105	[48]
10	***C***[b]	29	*anti*	Pd_2_(dba)_3_	dppe-C_6_H_6_[h]	–	toluene	*t*BuONa, ArBr[l]	105	[48]
11	***C***[b]	30	*syn*	Pd_2_(dba)_3_	(4-MeOC_6_H_4_)_3_P	–	toluene	*t*BuONa, ArBr^[l, m]^	105	[48]
12	***D***^[b,d]^	31	*syn*	PdCl_2_	MeCN	–	MeCN	–	RT	[50]
13	***E***[b]	32	*syn*	Pd(OAc)_2_	pyridine	–	-	calculations	RT	[51,52]
14	***E***[b]	33	*syn*	Pd(OAc)_2_	**125**[h]	O_2_	toluene	3 Å MS	25	[52,53]
15	***E***[b]	33	*anti*	Pd(O_2_CCF_3_)_2_	**125**[h]	O_2_	toluene	3 Å MS	25	[52,53]
16	***E***[b]	33	*syn*	Pd(O_2_CCF_3_)_2_	–	O_2_	toluene	3 Å MS	25	[52,53]
17	***E***[b]	34	*anti*	Pd(OAc)_2_	–	*p*-BQ	THF, DMSO	AcOH, AcONa	50	[54]
18	***F***[b]	38	*syn*	Pd(OAc)_2_	**139**[h]	–	dioxane	Cs_2_CO_3_, ArBr	105	[60,61]
19	***F***[b]	39	*syn*	Pd_2_(dba)_3_	(*t*Bu)_3_P	–	toluene	*t*BuONa, ArBr	65	[62]
20	***F***[b]	39	*syn*	Pd_2_(dba)_3_	Xanpthos[h]	–	toluene	*t*BuONa, ArBr	65 or 110	[62]
21	***F***^[b,d,e]^	40	*syn*	Pd_2_(dba)_3_	dppf[h]	–	THF	(Me_3_Si)_2_NK, ArBr[l]	23–60	[63,64]
22	***F***[b]	41	*syn*	Pd_2_(dba)_3_	Siphos-PE	–	xylene	*t*BuONa, ArBr	115	[65]
23	***F***[b]	42	*anti*	Pd(O_2_CCF_3_)_2_	–	(PhSO_2_)_2_NF[i]	toluene	ArOH, toluene	RT	[66]
24	***G***[b]	43	*syn*[g]	Pd(OAc)_2_	–	CuBr_2_	DMF	Na_3_PO_4_	40	[71]
25	***G***[b]	44	*syn*[g]	Pd(OAc)_2_	–	CuBr_2_	DMF	Na_3_PO_4_	40	[72]
26	***G***[b]	46	*anti*	Pd(O_2_CCF_3_)_2_	–	(PhSO_2_)_2_NF[j]	toluene	(PhSO_2_)_2_N^−^	RT	[66]
27	***H***[b]	47	*anti*	PdCl_2_	–	CuCl_2_	MeOH or AcOH	CO	RT	[75]
28	***H***[b]	49	*syn*	PdCl_2_	–	Cu(OAc)_2_	MeOH	CO, MeC(OMe)_3_	50	[77]
29	***I***[f]	13	*anti*	Pd(OAc)_2_	–	*p*-BQ	AcOH	AcONa	RT	[22]
30	***J***^[d,f]^	19	*anti*	PdCl_2_	MeCN	–	THF	Me_2_NH	−40	[31]
31	***K***[f]	50	*anti*	PdCl_2_	MeCN	PhI(OAc)_2_[k]	(CH_2_Cl)_2_	phthalimide, ArOH	70	[80]
32	***E***[f]	51	*syn*[n]	Pd(OAc)_2_	–	O_2_	(CH_2_Cl)_2_	phthalimide	70	[80]
33	***G***[f]	52	*anti*	PdCl_2_	PhCN	PhI(OAc)_2_[j]	(CH_2_Cl)_2_	phthalimide, Ts_2_NH	70	[85]

[a] ***A***=alkoxypalladation-carbonylation; ***B***=alkoxypalladation-HPdX elimination; ***C***=alkoxypalladation-arylation; ***D***=amidopalladation; ***E***=amidopalladation-HPdX elimination; ***F***=amidopalladation–arylation; ***G***=diamidopalladation; ***H***=amidopalladation-carbonylation; ***I***=acetoxypalladation; ***J***=aminopalladation; ***K***=amidoacetoxylation.

[b] Intramolecular reaction.

[c] Note that the reaction cannot proceed via 4(*O*)^*n*^-*exo-trig* cyclization.

[d] Stoichiometric reaction.

[e] The starting amide was first deprotonated.

[f] Intermolecular reaction.

[g] A different interpretation suggests *anti*-stereochemistry; see the comments in the text.

[h] Chelating ligand.

[i] Required in the second step, where the Pd^II^ intermediate **157** is oxidized to generate a Pd^IV^ species that then reacts with toluene via a C=H activation.

[j] Required in the second step for the oxidation **157** (Pd^II^) → **171** (Pd^IV^); the latter species then undergoes an S_N_2-type introduction of the second nitrogen group.

[k] Required in the second step for the oxidation **193** (Pd^II^) → **194** (Pd^IV^); the latter species then undergoes an S_N_2-type introduction of AcO.

[l] ArBr is connected to the C=C bond by a linker.

[m] Note the dynamic stereodifferentiaion by the residing chiral center.

[n] Only 5 % yield.

The intramolecular alkoxypalladation-carbonylation cascade with carbon monoxide and alcohol as the stoichiometric reactants (reaction ***A***) preferentially proceeds via the *anti*-pathway (entries 1–3), except for the examples, where this mechanism is disfavored by geometrical restrictions in the substrate (entry 4). On the other hand, *anti***/***syn* dichotomy has been observed for the analogous amidopalladation–carbonylation (reaction ***H***; entries 27 and 28). Here, the dramatic difference in the stereochemistry can be tentatively attributed to the difference in the pH (neutral vs acidic), which is likely to influence the coordination capabilities of the participating amidic group.

Stereochemistry of the intramolecular alkoxypalladation-β-elimination cascade (reaction ***B***; entries 5–7) can be controlled by the anion: thus, with PdCl_2_, that is, with strongly coordinating chlorides, especially in the presence of additional LiCl, the potential coordination of the participating alcohol group to Pd is disfavored and the reaction proceeds as an *anti*-addition (entry 5). On the other hand, with a weekly coordinating anion (BF_4_^−^ or CF_3_CO_2_^−^), Pd^II^ can become coordinated to the participating OH group, which results in the preferential *syn*-addition (entries 6 and 7), regardless of the oxidizing reagents or solvent.

The analogous intramolecular amidopalladation-β-elimination cascade (reaction ***E***; entries13–17) also exhibits the *syn*/*anti* dichotomy, depending on the actual conditions: thus, the reactions catalyzed by Pd(OAc)_2_ or Pd(O_2_CCF_3_)_2_ follow the *syn*-pathway (entries 13, 14, and 16); however, the presence of ligand **125** has been found to drive the reaction catalyzed by Pd(O_2_CCF_3_)_2_ toward the *anti-*mechanism (entry 15). By contrast, this ligand effect was not observed in the case of Pd(OAc)_2_ (entry14), which is rather intriguing. The change of the oxidizing agent (*p-*BQ vs O_2_) and the solvent (THF-DMSO vs toluene) and additives, namely AcOH/AcONa, has been found to also drive the reaction toward *anti*-mechanism (compare entries 14 and 16 with 17). Calculations predict the *syn*-mechanism (entry 13), which was also observed for the stoichiometric cyclization using PdCl_2_ (reaction ***D***; entry 12), where the palladated product was isolated.

The intramolecular alkoxypalladation-arylation cascade (reaction ***C***; entries 8–11) requires a strong base (as in the Hartwig-Buchwald arylation), which converts the participating alcohol group into a strongly coordinating alkoxide. As a result, the initial alkoxypalladation favors the *syn*-mechanism (entries 9 and 11). Addition of a chelating ligand may change the stereochemical course to *anti* (entry 10) but apparently not always (entry 8), especially when THF is used as a solvent (entry 8) instead of toluene (entry 10).

In analogy, the intramolecular amidopalladation-arylation cascade (reaction ***F***; entries 18–22), also occurring in the presence of a base, invariably proceeds as a *syn*-addition, regardless of the solvent or the nature of the ligand employed. On the other hand, the reaction that involves oxidation to Pd^IV^ in the second step (required for the C=H activation of the “nucleophile”) has been shown to proceed with *anti*-stereochemistry, even when catalyzed by Pd(O_2_CCF_3_)_2_ (entry 23). The outcome in the latter case can be attributed to the absence of the base, which renders the participating *N*-nucleophile less prone to coordination of the Pd^II^ catalyst.

Intramolecular diamidopalladation (reaction ***G***; entries 24 and 25) has been found to prefer the *syn*-mechanism, apparently due to the same effects as those discussed for entries 18–22. Again, this reaction, involving the Pd^II^ → Pd^IV^ oxidation and proceeding in the absence of a base, favors the *anti*-pathway (entry 26).

Intermolecular hydroxypalladation clearly prefers the *anti*-mechanism, as shown by the recent studies of the Wacker oxidation (Schemes 16–17). In analogy, intermolecular catalytic acetoxypalladation (reaction ***I***; entry 29) also follows the *anti*-pathway. The same stereochemistry has been demonstrated for the stoichiometric intermolecular aminopalladation with Me_2_NH (reaction ***J***; entry 30).

Intermolecular amidoacetoxylation (reaction ***K***; entry 31) and diamidopalladation (reaction ***G***; entry 33), catalyzed by PdCl_2_ (which disfavors coordination of the nucleophile to Pd), give the *anti*-addition products. By contrast, the intermolecular amidopalladation-β-elimination cascade (reaction ***E***; entry 32), catalyzed by Pd(OAc)_2_, favors the *syn*-mechanism, which seems to be the only experimentally proven example of *syn*-migration in intermolecular nucleopalladation with O- and N-nucleophiles (although only in 5 % yield) to date. Note that this reaction proceeds with a different oxidizing agent (O_2_) than those cited in entries 31 and 33, and that it does not involve the Pd^IV^ species. This, however is unlikely to have a major effect on the mechanism of the first step; it appears that the key point here is the use of Pd(OAc)_2_ rather than PdCl_2_ as the catalyst.

## 8. Conclusion

This review has discussed the stereochemistry of the palladium-catalyzed addition of nucleophiles to alkenes and application of these processes in organic synthetic transformations. The *syn*/*anti*-dichotomy in the nucleophilic additions was discussed, mainly with oxygen and nitrogen nucleophiles. A general picture is emerging that in intermolecular reactions the *anti*-addition of the oxygen and nitrogen nucleophiles to (alkene)Pd^II^ complexes is strongly favored. However, in intramolecular reactions a special situation arises when the nitrogen or oxygen nucleophile coordinates to Pd^II^. In this case there is no nucleophile available for external attack since there is a 1:1 ratio between the nucleophilic site and substrate. Therefore, the *syn*-attack is tremendously favored in the intramolecular cases where the nucleophile is coordinated to the metal. However, stereochemistry of the intramolecular reactions is dependent on the coordination capability of the internal nucleophile, which can be modified by the reaction conditions, so that the whole process can be driven either to the *syn*- or *anti*-pathway. In the intermolecular process, there will always be a considerable amount of free nucleophile in solution and therefore coordination of the nucleophile does not shut down the external *anti*-pathway as is done in the intramolecular case. As a result, external *anti*-attack is the predominant pathway in the intermolecular nucleophilic addition to (alkene)Pd^II^ complexes.
